# Using a combination of quantitative culture, molecular, and infrastructure data to rank potential sources of fecal contamination in Town Creek Estuary, North Carolina

**DOI:** 10.1371/journal.pone.0299254

**Published:** 2024-04-19

**Authors:** Jenna M. Hynes, Rachelle E. Beattie, A. Denene Blackwood, Thomas Clerkin, Javier Gallard-Góngora, Rachel T. Noble

**Affiliations:** Department of Earth, Marine and Environmental Sciences, Institute of Marine Science, University of North Carolina at Chapel Hill, Morehead City, North Carolina, United States of America; Cranfield University, UNITED KINGDOM

## Abstract

Estuarine water quality is declining worldwide due to increased tourism, coastal development, and a changing climate. Although well-established methods are in place to monitor water quality, municipalities struggle to use the data to prioritize infrastructure for monitoring and repair and to determine sources of contamination when they occur. The objective of this study was to assess water quality and prioritize sources of contamination within Town Creek Estuary (TCE), Beaufort, North Carolina, by combining culture, molecular, and geographic information systems (GIS) data into a novel contamination source ranking system. Water samples were collected from TCE at ten locations on eight sampling dates in Fall 2021 (n = 80). Microbiological water quality was assessed using US Environmental Protection Agency (U.S. EPA) approved culture-based methods for fecal indicator bacteria (FIB), including analysis of total coliforms (TC), *Escherichia coli* (EC), and *Enterococcus spp*. (ENT). The quantitative microbial source tracking (qMST) human-associated fecal marker, HF183, was quantified using droplet digital PCR (ddPCR). This information was combined with environmental data and GIS information detailing proximal sewer, septic, and stormwater infrastructure to determine potential sources of fecal contamination in the estuary. Results indicated FIB concentrations were significantly and positively correlated with precipitation and increased throughout the estuary following rainfall events (p < 0.01). Sampling sites with FIB concentrations above the U.S. EPA threshold also had the highest percentages of aged, less durable piping materials. Using a novel ranking system combining concentrations of FIB, HF183, and sewer infrastructure data at each site, we found that the two sites nearest the most aged sewage infrastructure and stormwater outflows were found to have the highest levels of measurable fecal contamination. This case study supports the inclusion of both traditional water quality measurements and local infrastructure data to support the current need for municipalities to identify, prioritize, and remediate failing infrastructure.

## Introduction

Estuaries provide a variety of recreational, economic, and ecosystem services to the populations that surround and inhabit their waters. Acute and chronic contamination events in estuaries are becoming more prevalent and often stem from a combination of natural and human-driven events. A common but problematic contaminant in estuaries is fecal waste, which frequently increases in concentration following storm events from stormwater runoff [1,2]. However, chronic fecal contamination in estuaries may often be a result of aging sewer structures, which can become overwhelmed due to infiltration, exfiltration, and inflow from increased use during seasonal tourism and due to weather including both typical precipitation conditions and extreme events such as hurricanes and tropical storms [3–8]. In an effort to mitigate contamination, coastal municipalities are increasingly using stormwater control measures to reduce estuarine contamination, particularly for nutrients, sediment, and fecal pollution [8–10]. However, stormwater control measures cannot account for the entirety of infrastructure-related contamination impacting estuaries. Extensive work along the coastal plain states of North Carolina and Virginia indicates that municipalities are seeking decision-making data that inform the prioritization of infrastructure repairs to reduce estuarine water quality degradation [11].

Sewage and stormwater infrastructure in the United States (US) have been highlighted in recent years as being aged and in dire need of repair [11,12]. Water infrastructure improvements are considered a priority by the US government, and funding for improvement projects was included in the passage of the 2021 Infrastructure Investment and Jobs Act [12]. This law has resulted in the federal government awarding more than 50 billion USD to the US Environmental Protection Agency (U.S. EPA) to distribute to states, Tribes, and territories to improve water infrastructure including sewage systems [13]. In February 2023, North Carolina Governor Roy Cooper approved 462.9 million USD in funding for 249 infrastructure projects in 80 communities state-wide [14]. Many of these funds have been earmarked for specific large-scale aging and remediation projects in metropolitan centers, but there is an opportunity to develop infrastructure improvements in smaller coastal regions receiving large numbers of recreational water users [15,16].

Town Creek Estuary (TCE) is a popular recreational estuary located in Beaufort, North Carolina, a small coastal town with a population of approximately 4,500 full time residents. Despite its small size, the Town of Beaufort and surrounding Carteret County see an annual visitor count exceeding one million individuals due to the abundance of water-related recreational activities in the region (15). Although tourism drives the local economy, coastal development required to support visitors has increased stress on aging sewer infrastructure and area septic systems, which in turn threatens estuarine ecosystems and water quality [1,10]. Poor estuarine water quality increases the potential risk of human exposure to harmful contaminants during recreation, which is critical in places such as Beaufort that rely on tourism. Waters contaminated with fecal material are estimated to cause 170 million enteric and respiratory illnesses annually [17].

The Town of Beaufort has a long history of collaborating with local researchers to identify contamination and develop mitigation strategies to preserve water quality [1,10,18,19]. From these studies and collaborations with the town, we have learned that Beaufort has an extensive underground network of sewer and stormwater infrastructure, with sewer pipe construction dating back to 1969. Sewer pipe material ranges from cured-in-place pipe (CIPP), ductile iron pipe (DIP), polyvinyl chloride (PVC), truss, or vitrified clay (VC). Studies have shown that sewer pipe durability declines with age with exponential declines in durability and increases in corrosion occurring by 50 years [4–6]. Moreover, the town has several stormwater outflows that drain into TCE. Stormwater runoff has been a consistent non-point source of microbial contamination nationwide [20]. High levels of biological contaminants, including fecal indicator bacteria (FIB) have been directly related to disease outbreaks in recreational swimming areas [3,7,18]. With multiple potential sources of fecal contamination in TCE, a thorough analysis of the estuary is crucial to protect water quality, preserve the local economy, and ensure public safety.

The primary objective of this study was to design an estuarine water quality monitoring program and establish the use of a ranking approach for identifying locations of potentially compromised stormwater and sewage infrastructure in a coastal town. To accomplish this, we developed a creek-estuary sampling transect across ten sites spanning from creek headwaters to a prominent recreation use location (junior sailing camp, maritime museum, and marina) and used GIS data to identify the infrastructure along the transect. We then integrated infrastructure data with water quality and microbial data to develop a ranking approach to prioritize potential areas in need of review and repair. This comprehensive approach of combining microbial source tracking, water quality data, and infrastructure data can be used by town managers to prioritize infrastructure and stormwater projects to improve estuarine water quality.

## Materials and methods

### Sample site selection

**Fig 1:**
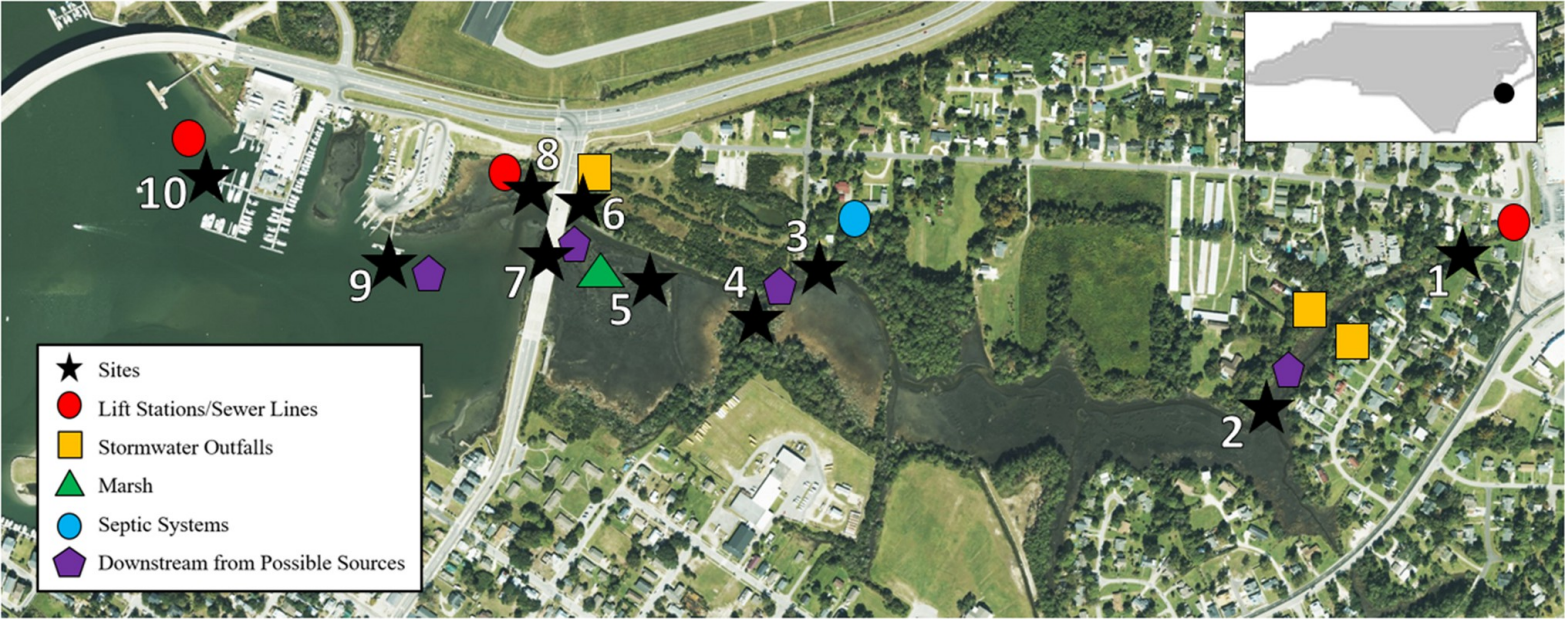
A map of Town Creek Estuary in Beaufort, NC, with the sampling sites denoted with a black star icon. Lift stations and nearby sewer infrastructure are denoted by a red circle icon; stormwater outfalls are denoted by a yellow square icon; marsh is denoted by a green triangle icon; septic systems are denoted by a blue circle icon; and areas downstream from possible major sources of contamination are denoted by a purple trapezoid icon.

TCE is located within the city limits of Beaufort, North Carolina, and encompasses approximately 0.36 square kilometers. The estuary provides a variety of water recreation activities including boating, fishing, and swimming, and is host to a summer sailing camp. To assess fecal contamination along the estuary and creek headwaters, ten sampling sites were selected (Table 1 and Fig 1). Sites were selected due to variable proximity to a) stormwater outfalls and sewer infrastructure, b) septic systems, c) lift stations, d) marinas, e) estuarine marsh, and f) down-channel from possible contamination sources. Site 5, located within the estuarine marsh, was used as a control because marsh habitat is known to naturally filter and attenuate contaminants [21–23].

**Table 1:** Sampling site locations in Town Creek Estuary and associated potential sources of contamination.

Site Number	Latitude	Longitude	Nearby Potential Sources
**1**	34.72564	-76.64764	Stormwater outflows, Sewer infrastructure
**2**	34.72326	-76.65102	Stormwater outflows, Down-channel from site 1
**3**	34.72486	-76.65752	Septic Systems
**4**	34.7247	-76.65756	Septic Systems, Down-channel from site 3
**5**	34.72464	-76.65914	Estuarine marsh
**6**	34.72566	-76.66037	Stormwater outflows
**7**	34.72552	-76.66073	Stormwater outflows, Down-channel from site 6
**8**	34.72583	-76.66103	Lift Station
**9**	34.72508	-76.66319	Marinas
**10**	34.72614	-76.66523	Lift Stations, Marinas

### Sample collection

Grab samples were collected in acid-washed 1 L HDPE bottles (ThermoFisher Scientific, Waltham, MA) at each of the ten sites immediately following high tide. Samples were collected at high tide to capture the flushing of potential contaminants from non-point sources. Each bottle was washed with the surrounding water three times before a sample was taken from an undisturbed section of water [1,10]. Samples were collected on eight dates between August 6, 2021, and October 11, 2021. At the location where each grab sample was collected, a YSI-EXO2 multi-parameter water quality sonde (YSI Inc./Xylem Inc., Yellow Springs, OH) was deployed just below the surface of the water, ensuring all probes were completely submerged, to measure water temperature (˚C), atmospheric pressure (mmHg), dissolved oxygen (%), specific conductance (μS-cm), salinity (ppt), and turbidity (FNU). Samples were held at ambient temperature until they were transported to the laboratory for processing. All samples were processed within two hours of collection. Using a dissolved oxygen conversion calculator produced by the University of Minnesota Natural Resources Research Institute and approved by the U.S. EPA, dissolved oxygen saturation was converted to mg/L [24].

### Local environmental conditions

Tide height was recorded in meters using daily historical tide charts measured at the Duke Marine Lab, station id: 8656483, in Beaufort, NC, maintained by the National Oceanic and Atmospheric Administration (NOAA) [25]. The base tide height was calculated by taking the average of all the high tide data provided by NOAA at the Duke Marine Lab from the first to the last sampling date. Any collection date with a tide height higher than the base represents a day with a larger-than-normal tidal influence. The previous 24 and 72 hours of precipitation in centimeters (cm) was determined by referencing reports gathered by the Community Collaborative Rain, Hail, and Snow Network at site NC-CR-139: Beaufort 0.5 W [26]. Any sampling date with measurable precipitation during both 24 hours and 72 hours prior was deemed a wet weather event (Table 2). Both precipitation measurements were used as several studies have shown that precipitation three days prior to sample collection has an impact on the concentration of microorganisms [2,27,28]. Wind speed and direction were collected from NOAA’s meteorological observation dataset recorded at the Duke Marine Lab, station id: 8656483, in Beaufort, NC [25]. Wind speed was reported in meters per second (m/s) and the wind direction was reported in degrees from true North. The wind direction was then converted to a cardinal direction by referencing the conversion chart provided by the University of Northern Iowa [29]. The base wind speed and direction were calculated by averaging the available data for both parameters from the first sampling date to the last. Any sampling date with a wind speed higher than the base represents a day with a larger-than-normal wind influence. Environmental condition data is available in Table 2.

**Table 2:** Average environmental conditions observed on each sampling date.

Sampling Date	Previous 24hr Precipitation (cm)	Previous 72hr Precipitation (cm)	Tide Height (m)	Wind Speed (m/s)	Wind Direction	Wet/Dry Event*
**8/6/2021**	0.28	15.37	0.85	2.05	NNE	Wet
**8/13/2021**	0.00	0.00	1.10	2.87	WSW	Dry
**8/27/2021**	0.00	2.50	1.07	2.84	SSW	Dry
**9/10/2021**	0.28	1.35	1.25	5.16	NNE	Wet
**9/20/2021**	0	2.90	1.13	1.99	NE	Dry
**9/24/2021**	3.56	4.20	1.16	5.51	N	Wet
**9/28/2021**	0.00	0.00	1.07	2.53	W	Dry
**10/11/2021**	1.09	5.31	1.31	5.31	N	Wet

Note: The base tide height is 1.07 m, the base wind speed is 3.42 m/s, and the base wind direction is SSE. *Denotes which sampling dates were considered wet or dry weather events for statistical analysis.

### Culture-based methods for determining FIB concentration

Colilert-18® and Enterolert ™ kits from IDEXX Laboratories (Westbrook, ME) were used to assess the most probable number (MPN) of total coliforms (TC), *Escherichia coli* (EC), and *Enterococcus spp*. (ENT) bacteria in each water sample following the manufacturer’s instructions. Briefly, a 1:10 dilution of each water sample was prepared by combining 90 mL of deionized water, 10 mL of raw sample, and respective test media into 120 mL bottles containing sodium thiosulfate (non-fluorescing polystyrene, IDEXX). The bottles were mixed by inversion for 30–60 seconds until fully homogenized and then placed on the bench top for a minimum of three minutes to ensure no bubbles were present. The solutions were then poured into individual IDEXX Quanti®-Tray 2000’s and sealed using a Quanti® - Tray Sealer PLUS (IDEXX). The trays containing Colilert-18® media were incubated for 18 hours at 35°C while the trays with Enterolert™ media were incubated for 24 hours at 41°C. After the appropriate incubation period, the trays were removed and analyzed. After quantifying the number of positive wells for each tray and assay, the MPN per 100 mL and 95% confidence interval were generated for TC, EC (S3 Table), and ENT (S4 Table) using the IDEXX MPN calculator. A solution of 1X PBS was prepared as a method blank and analyzed with each batch of samples that were processed to ensure there was no contamination during processing. Each grab sample was tested in duplicate, and the MPN values that were generated from each Quanti®-Tray 2000 were averaged to generate a mean value for each sampling site for each sampling event.

### DNA extraction and droplet digital PCR (ddPCR) for quantitative microbial source tracking (qMST)

Water samples collected in the field were filtered for DNA within two hours of collection. Duplicate 150 mL water samples were vacuum filtered to dryness using 47 mm diameter polycarbonate filters with a 0.4 μm pore size (HTTP, Millipore, Bedford, MA) on a six-place filtration manifold, and vacuum pump apparatus. Following filtration, filters were placed into DNase/RNase free microcentrifuge tubes using sterile forceps and stored at -80°C until downstream processing for a maximum of 3 months. DNA was extracted from one filter per sample following lysis with 1 mL easyMag® Lysis buffer (BioMérieux, Durham, NC) containing 7.4x10^5^ copies of the extraction recovery control, *gyrA*, from a haloalkaliphilic archaeon. The lysed samples were incubated for 10 minutes at room temperature followed by total nucleic acid extraction using an automated magnetic particle analyzer (KingFisher™Flex, Thermo Fisher Scientific, Waltham, MA) and easyMag® NucliSENS® reagents (BioMérieux, Durham, NC), and eluted in 100 μL of Buffer AE (19077, QIAGEN, Germantown, MD). Specific details for the automatic extraction method can be found in Beattie *et al*. 2022 [30].

A duplexed PCR mastermix targeting HF183 and the *gyrA* gene were created by adding 0.9 nM of the respective forward and reverse primers, 0.25 nM of the respective probes, 12.5 μL of ddPCR™ 2X Supermix for Probes (nodUTP, Bio-Rad Laboratories), 5 μL of DNA, and nuclease free water to a final reaction volume of 25 μL. Each sample was run in duplicate. Primers and probes were purchased from LCG Bioresearch (Petaluma, CA), and the sequences are shown in Table 3 for the HF183 assay [31,32]; sequences for the haloalkaliphilic archaeon *gyrA* assay were kindly provided by John Griffith (Southern California Coastal Water Research Project, Costa Mesa, CA) and are in preparation for publication [33]. In this study, *gyr*A was spiked into the extraction buffer and was used to assess both extraction recovery and inhibition. Positive HF183 controls (see S1 Method), no-extraction controls, and no template controls were included with each assay plate in addition to method blanks from each sampling date. No extraction controls consisted of a sterile 47 mm polycarbonate filter (0.4 μM pore size) extracted using the same method as sample filters. These samples were then analyzed using ddPCR using the same method as sample filters. No-template controls consisted of PCR master mix containing nuclease free water instead of sample DNA. Additional MIQE details can be found in S9 Table.

**Table 3:** Primers and probes for the HF183 assay [32].

Assay	Oligo ID	Sequence	Concentration	Position number*	Reference
**HF183 TaqMan**	HF183	ATCATGAGTTCACATGTCCG	0.9 μM	180–199	Haugland et al. (2010)
	BFDRev	CGTAGGAGTTTGGACCGTGT	0.9 μM	346–327	
	FAM	CTGAGAGGAAGGTCCCCCACATTGGA	0.25 μM	295–319	

*Sequence for position number obtained from the 16S rRNA gene of *Bacteroides dorei* strain 175, NR_041351.1.

Twenty μL of the PCR mastermix and sample were pipetted into sample wells of the DG8™ Cartridge (Bio-Rad,) using a manual 8-channel pipette (L8-50XLS+, Rainin, Oakland, CA) followed by the addition of 70 μL of Droplet Generation Oil for Probes (Bio-Rad) to the oil wells. The cartridges were covered with DG8™ Gaskets (Bio-Rad) and processed in a manual Droplet Generator (Bio-Rad). The droplets were gently transferred to a semi-skirted 96-well PCR plate (mTEC, Eppendorf, Framingham, MA) using a manual 8-channel pipette. The PCR plate was sealed with pierceable foil (Bio-Rad) using a PX1™ PCR Plate Sealer (Bio-Rad).

The PCR plate was placed in a C1000 Touch™ Thermal Cycler (Bio-Rad) and amplification was performed with the following temperature profile: 10 min at 95°C for initial denaturation, 40 cycles of 95°C for 30 s, and 58°C for 60 s with a ramp rate of 2°C/s, followed by 98°C for 10 min, then an indefinite hold at 4˚C (Zhu et al. 2020). After PCR cycling was complete, the plate was placed in a QX200™ instrument (Bio-Rad) and droplets were analyzed according to manufacturer’s instructions for 6-FAM™/HEX™. Data acquisition and analysis were performed with QuantaSoft™ v. 1.7 (Bio-Rad). The fluorescence amplitude threshold, distinguishing positive from negative droplets, was set manually by the analyst at the midpoint between the average baseline fluorescence amplitude of the negative droplet cluster and the positive droplet cluster (S1 Fig). The same threshold was applied to all the wells of one PCR plate. Measurement results of single PCR wells were excluded if the total number of accepted droplets was <10,000 or the average fluorescence amplitudes of positive or negative droplets were clearly different from those of the other wells on the plate, in accordance with manufacturer guidelines. The QuantaSoft software uses the Poisson distribution to quantitate the concentration of targets based on the numbers of positive and accepted droplets in each well. Samples were quantified in duplicate using S1 Equation and replicate wells were merged. A sample was considered positive and quantifiable if the minimum threshold of three positive droplets was met.

### Geographic information systems

All analysis on sewer and stormwater infrastructure was done using ArcGIS Pro 2.7.0 (Esri Inc., Redlands, California). Shapefiles of the sewer and stormwater infrastructure were provided by the Beaufort, NC, Town Engineer (2021). The attribute table of each shapefile included information on each pipe’s diameter, length, material, and date of construction. The attribute tables also included the location and date of construction of nearby lift stations and stormwater outflows. The projected coordinate system used for all maps was NAD 1983 ft US and all shapefiles were overlaid on the Hybrid Reference Layer base map (Esri Inc, Redlands, California). All mapping figures were made using National Agriculture Imagery Program (NAIP) aerial imagery from the United States Geological Society Earth Resources Observatory and Science (USGS EROS) database.

### Models for ranking sources of fecal contamination

Two models were developed to rank potential sources of fecal contamination in TCE: an equal weight model and a variable weight model. Six parameters were included from each site: mean EC concentration, mean ENT concentration, mean HF183 concentration, percentage of pipes in a 400-m radius made of vitrified clay, percentage of pipes in a 400-m radius over 50 years of age, and the inverse of the distance to the nearest stormwater pipe in a 400-m radius. The parameters were ranked at each site, with 10 being the highest concentration, percentage, or inverse distance of the parameter and 1 being the lowest. For the equal weighting model, each parameter was given equal weight, thus the overall site ranking was based on the total sum of the rank of each parameter at each site. See S2 Method for additional details.

For the variable weighting model, empirical knowledge was used to weight parameters based on whether or not the parameter indicated the presence of fecal contamination; i.e., measured markers of fecal contamination (FIB, EC, ENT, HF183) were assigned a higher weight. Those parameters directly linked to fecal contamination (such as HF183 and FIB concentrations) were given a higher weight in the model. Parameters were weighted 1–6, with 6 being the highest weight, based on whether or not the presence of the parameter indicated the presence of human fecal contamination, with those parameters directly linking fecal contamination to human sources ranked the highest weight. If a parameter had an equal likelihood of indicating the presence of fecal contamination as another parameter, they were given the same weight and the values of subsequent weight were adjusted accordingly; see the weights given to each parameter using the variable weight model in S2 Method.

For both models, each parameter weight was multiplied by the parameter rank at each site, and a value was calculated. The value of the six parameters at each site was summed, and the site with the highest total sum was considered the highest potential source of fecal contamination, represented by a final rank of 1. Additional details can be found in S2 Methods.

### Statistical analysis

Any quantified values for FIB MPN per 100 mL that were below and above detection limits containing a “<” or “>” symbol, as assigned by the IDEXX MPN calculator, were reassigned with the next respective numerical value for use in statistical calculations [1,34]. For example, a value of “<10” was changed to 9 and a value of “>24196” was changed to 24197. Additionally, all non-detect HF183 concentrations were scored as 0 for statistical analyses. All FIB and microbial gene marker concentration data were log_10_ transformed to reduce the skewness as the Shapiro-Wilks test determined the data was not normally distributed (p < 0.05). All statistical tests were performed at a significance level of p = 0.05 and a confidence level of 95%. Differences between site concentrations of the log_10_ transformed FIB, molecular qMST targets, and environmental parameters were determined by using Kruskal-Wallis test for non-parametric data. If significant differences were identified, the Dunn test was used to determine which collection sites and dates differed significantly. The non-parametric Spearman’s rank correlation test was used to evaluate correlations between FIB, microbial gene marker, environmental data, and sewer infrastructure; the strength of the correlation is denoted by the Spearman’s rank correlation coefficient, r_s_. All statistical tests were conducted using R software (R Core Team, Vienna, Austria) [35] in RStudio (Rstudio Team, Boston, MA) using the tidyverse package [36], dpylr package [37], car package [38], corrplot package [39], and the lme4 package [40]. All figures were created using the ggplot2 package [41].

## Results

### Water quality

**Fig 2:**
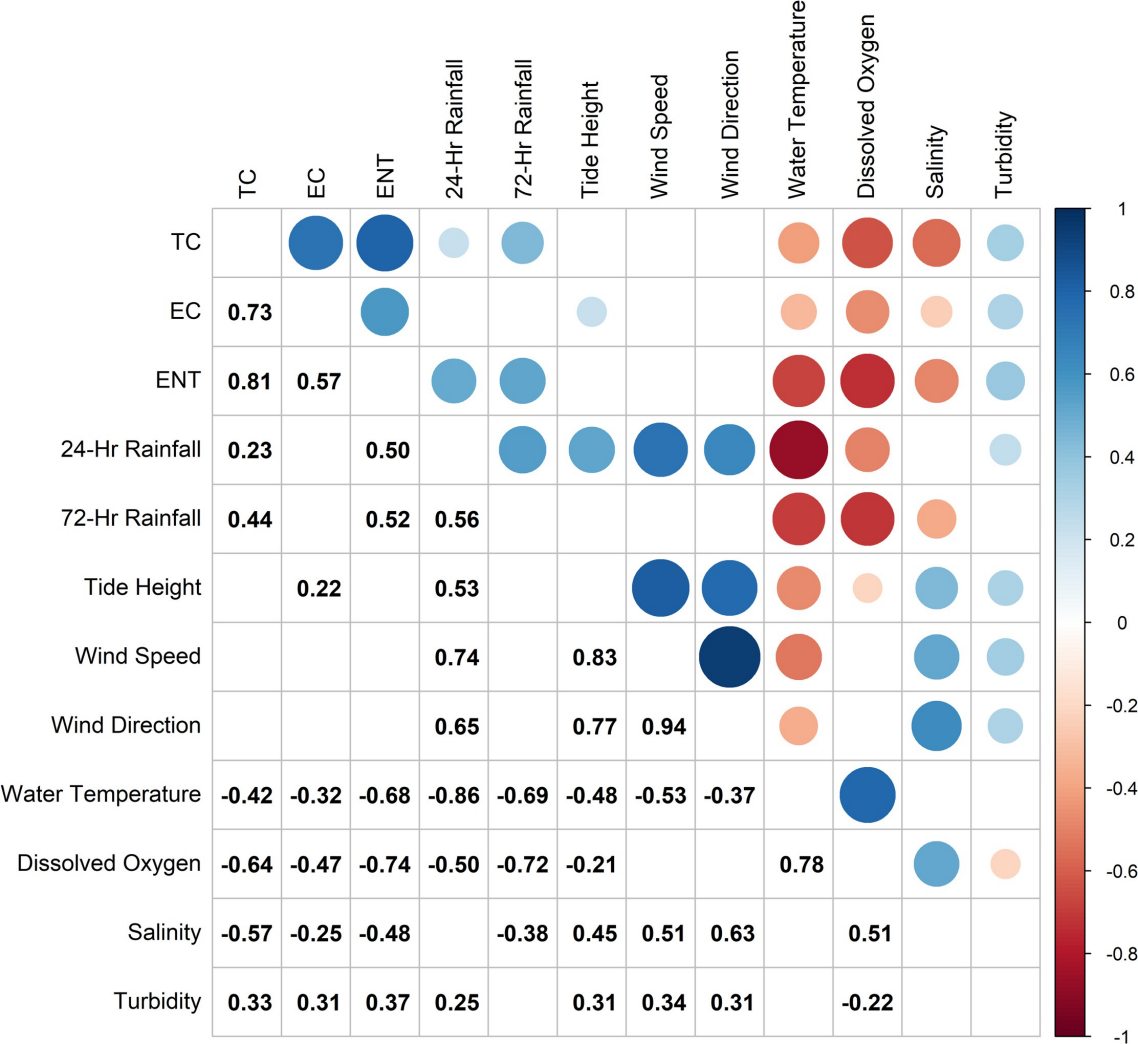
Correlation plot of Spearman rank correlations between environmental, water quality, and molecular parameters. Significant (p < 0.05) correlations are shown on the upper portion of the plot as red dots (negative correlations) or blue dots (positive correlations) with the correlation strength represented by the shade of the dot. The size of the dot indicates the significance level with larger dots representing a higher probability that the identified correlation is real. The lower portion of the plot shows the spearman rank correlation values. TC = total coliforms, EC = *Escherichia coli*, ENT = *Enterococcus*.

Water quality parameters, such as salinity, dissolved oxygen, and temperature, varied throughout the TCE and were positively associated with precipitation patterns (n = 80, Fig 2 and S1 Table). Channel salinity (ppt) ranged from 0.2 in creek headwaters (Site 1) to 36.4 in the open channel (Site 10). Salinity differed significantly by site (p < 0.05) under all conditions. Dissolved oxygen levels were lower at sites within creek headwaters (Site 1, mean saturation = 43.3%, equivalent to 3.6 mg/L) compared to the open channel (Site 10, mean saturation = 82.2% equivalent to 6.6 mg/L) over the 10-week study period. Water temperature was more stable across sites, with limited variability over the 10 weeks study (min. 24.5°C recorded at Site 1 and max 27.6°C recorded at Site 5).

*Enterococcus spp*. concentrations ranged from 9 to 24,197 MPN per 100 mL (95% CI [0, 37] and [16304, 47161] respectively), and EC concentrations ranged from 77.5 to 24,197 MPN per 100 mL (95% CI [32.5, 146.0] and [NA, Infinite] respectively) across all sites over the course of the study (S3 and S4 Tables). The highest measured concentrations of FIB were observed in creek headwaters and locations adjacent to stormwater drains while lower concentrations were detected in the open, deeper, more tidally influenced reaches of the estuary. For example, Site 1 in the creek head waters had high FIB concentrations with ENT between 260.5 to 24,196.5 MPN per 100 mL (95% CI [162.5, 392.5] and [16304, 47161] respectively) and EC between 381.5 to 6,330.5 MPN per 100 mL (95% CI [257, 547] and [4142, 9108] respectively). In contrast, Site 10 in the open channel had ENT concentrations ranging from 9 to 20.5 MPN per 100 mL (95% CI [0, 37] and [4, 72] respectively) and EC concentrations ranging from 86.5 to 459 MPN per 100 mL (95% CI [41.5, 155.5] and [323, 630.5] respectively, S2–S4 Tables).

Fecal indicator bacteria concentrations trended higher across the transect after wet weather events (n = 40) when compared to dry weather (n = 40). Precipitation patterns were found to significantly and positively correlate with measured concentrations of ENT (previous 24 hours: r_s_ = 0.50, p < 0.001 and previous 72 hours: r_s_ = 0.52, p < 0.001), with mean concentrations of ENT following a wet weather event equal to 1,053.1 MPN per 100 mL (sd = 3877.4 MPN per 100 mL) compared to dry weather with mean concentrations of 104.4 MPN per 100 mL (sd = 299.4 MPN per 100 mL). The mean concentration of ENT during wet weather events exceeded the U.S. EPA and North Carolina Department of Environmental Quality (NCDEQ) recreational water quality standard of 104 MPN per 100 mL [42]. *Enterococcus* concentrations were found to significantly and positively correlate with wet weather events (p <0.01). Additionally, ENT concentrations were significantly and negatively correlated with salinity (r_s_ = -0.48, p < 0.001), dissolved oxygen (r_s_ = - 0.74, p < 0.001), and water temperature (r_s_ = - 0.68, p < 0.001) (Fig 2 and S5A and S5B Table).

In contrast to ENT concentrations, EC exceeded the U.S. EPA recreational water quality standard of 320 MPN per 100 mL [42] during both wet and dry conditions with a wet weather mean concentration of 2644.7 MPN per 100 mL (sd = 6364.3 MPN per 100 mL) and a dry weather mean of 795.6 MPN per 100 mL (sd = 1404.6 MPN per 100 mL) across sites. Of the total samples collected during wet weather events (n = 40), 23 (57.5%) exceeded the U.S. EPA standard for EC and 22 (55%) exceeded the U.S. EPA standard for ENT compared to dry weather when 18 (45%) exceeded the EC standard and 5 (12.5%) exceeded the ENT standard. *E. coli* concentrations also had a significant and negative correlation with salinity (r_s_ = -0.25, p < 0.05), dissolved oxygen (r_s_ = -0.47, p < 0.001), and water temperature (r_s_ = -0.32, p < 0.05), although these correlations were not as strong as with ENT concentrations (Fig 2 and S5A and S5B Table). Additionally, there was no significant relationship between wet/dry weather events and EC concentrations. No FIB species were detected in any of the method blanks.

### Quantitative microbial source tracking

To identify if the source of fecal contamination observed in the TCE was of human origin, the human host-associated marker HF183 was measured in all samples and eight field blanks (one per sampling event). HF183 was detected in six of 80 samples (7.5% detection rate) with four of those detections following wet weather events; HF183 was not detected in any field blank (S6 Table). Concentrations ranged from a mean of 33.5 copies per 100 mL during dry weather (sd = 7.4 copies per 100 mL) to 108.3 copies per 100 mL during wet weather (sd = 36.9 copies per 100 mL); however, overall detection in the estuary was low. Each assay plate included three no extraction controls, three no template controls, and three positive controls in addition to the method blank samples from each collection event. HF183 was detected in each positive control and was not detected in any negative control or method blank, as expected. Fluorescence plots did not indicate assay inhibition in study samples due to the lack of partial amplification (rain) and the tight clustering of positive fluorescent signal observed in our fluorescence plots (S1 Fig) [43].

### Sewer and stormwater infrastructure

Stormwater and sewer pipe age, material type, and location were assessed using GIS data provided from the Town of Beaufort, NC. The GIS infrastructure layers were updated in the Town of Beaufort in Fall of 2021, providing an excellent opportunity to use the most updated information for this study. Beaufort, NC, has approximately 53,000 meters of underground sewer pipe. Along the perimeter of the TCE (approximately 400 meters), most sewer pipes were constructed in 1969 (52.3%) and 2008 (32%). Pipe material throughout the Town of Beaufort includes CIPP, DIP, PVC, truss, and VC. The VC pipes were all constructed in 1969. Around the perimeter of the TCE, most pipes are constructed out of VC (30.8%) and CIPP (25%). Additionally, there is one lift station adjacent to site 8 and another adjacent to site 10 (Fig 1). In addition to sewer pipe, there are approximately 55,000 meters of underground stormwater pipe that span the town including around the perimeter of TCE. Stormwater infrastructure data indicated three of 71 total discharge points are located within TCE (Fig 1). Distance between sites and stormwater outflows in the TCE ranged from five to 400 meters.

### Integrating FIB, qMST, and GIS-based infrastructure data

A major goal of this project was to integrate stormwater and sewer infrastructure information with water quality data to develop a ranking system to identify potential areas in need of assessment, remediation, or structural testing [44]. TCE is an ideal case study location for this analysis as the estuary is geographically small, highly valuable for recreational water quality activities, well-studied [18], and the town has detailed information about the infrastructure surrounding the site. In order to rank the samples sites as potential sources of fecal contamination, several factors were considered. First, the mean concentrations of EC, ENT, and HF183 at each site were used as the biological parameters. Next, the infrastructure data within a 400-meter radius of each site was calculated including the percentage of sewer pipes aged over 50 years, the percentage of sewer pipes made of VC, and the inverse of the distance to the nearest stormwater outflow. Two different models were used to assess potential fecal contamination sources using the six parameters, 1) an equal weighting of each parameter, and 2) a variable weight of each parameter with a higher weight given to those parameters that explicitly link fecal contamination to the site (EC, ENT, HF183). Details of the two models can be found in the methods and supporting information, S2 Method.

*Enterococcus spp*. and EC concentrations were significantly different between sites (p < 0.001) with Sites 1 and 2 having higher concentrations as compared to other sites across the estuarine transect. Mean ENT concentrations varied from a low of 12.3 (Site 10) to a high of 4504.3 MPN per 100 mL (Site 1, Table 4). Of the 10 sites, four had mean ENT concentrations above recreational water quality standards [42] and three of those sites were located in TCE headwaters. Mean EC concentrations varied from a low of 200.4 (Site 9) to a high of 6,429.6 (Site 6) MPN per 100 mL (Table 4). Even though the State of NC does not utilize EC for recreational water quality management, we measured EC in this study because of its prominent use in other coastal states for recreational water quality management. Of the 10 sites, six had mean EC concentrations above the U.S. EPA standard of 320 MPN per 100 mL [42]. Four of those seven sites (Sites 1, 2, 6, 7) had mean EC concentrations over 2,000 MPN per 100 mL. Although there was limited detection of the qMST marker, HF183, the data was included in the site ranking due to its direct link to human fecal contamination and subsequent linkage to human health (e.g. Boehm et al. 2015 [45]).

**Table 4:** Mean values of vitrified clay (VC) pipe and pipes aged over 50 years in a 400-meter radius of each site; mean concentrations of *Escherichia coli* (EC), *Enterococcus* (ENT), HF183, and distance to the nearest stormwater pipe within a 400-meter radius of each site. Ranks were assigned using two models, equal weighting of each parameter or variable weighting with a higher weight assigned to parameters of measured fecal contamination. A rank of 1 indicates the highest potential source location of fecal contamination and priority for infrastructure review and repair to 10 with the lowest potential source risk. Bold values indicate equivalent ranks using both models.

Site	Vitrified Clay Pipes (%)	Pipes aged over 50 years (%)	Mean EC (MPN per 100 mL)	Mean ENT (MPN per 100mL)	Mean HF183 (Copies per 100mL)	Approximate Distance to Nearest Stormwater Pipe (m)	Rank Using Equal Weighting	Rank Using Variable Weighting
1	32%	64%	2907.6	4504.3	42.0	60	**1**	**1**
2	47%	65%	2588.9	1093.9	5.0	80	**2**	**2**
3	29%	37%	513.4	284.7	0.0	100	4^#^	5
4	26%	33%	308.4	71.5	4.6	100	6	3^#^
5	26%	33%	282.4	77.4	0.0	60	7	8
6	19%	25%	6429.6	256.4	0.0	5	**3**	**3** ^#^
7	19%	25%	3331.0	69.4	0.0	15	4^#^	6
8	14%	18%	404.2	57.8	0.0	20	8	9
9	19%	19%	200.4	37.4	0.0	95	9	10
10	0%	0%	235.3	12.3	10.9	160	10	7

^#^Using the equal weighting method, Sites 3 and 7 were equally ranked as the 4^th^ most likely to contribute to fecal contamination in the estuary; using the variable weighting method, Sites 3 and 6 were equally ranked as the 3rd most likely to contribute to fecal contamination in the estuary. The remaining site rankings were adjusted accordingly (i.e., the next highest ranked site received a score of 6 and 5, respectively).

**Fig 3:**
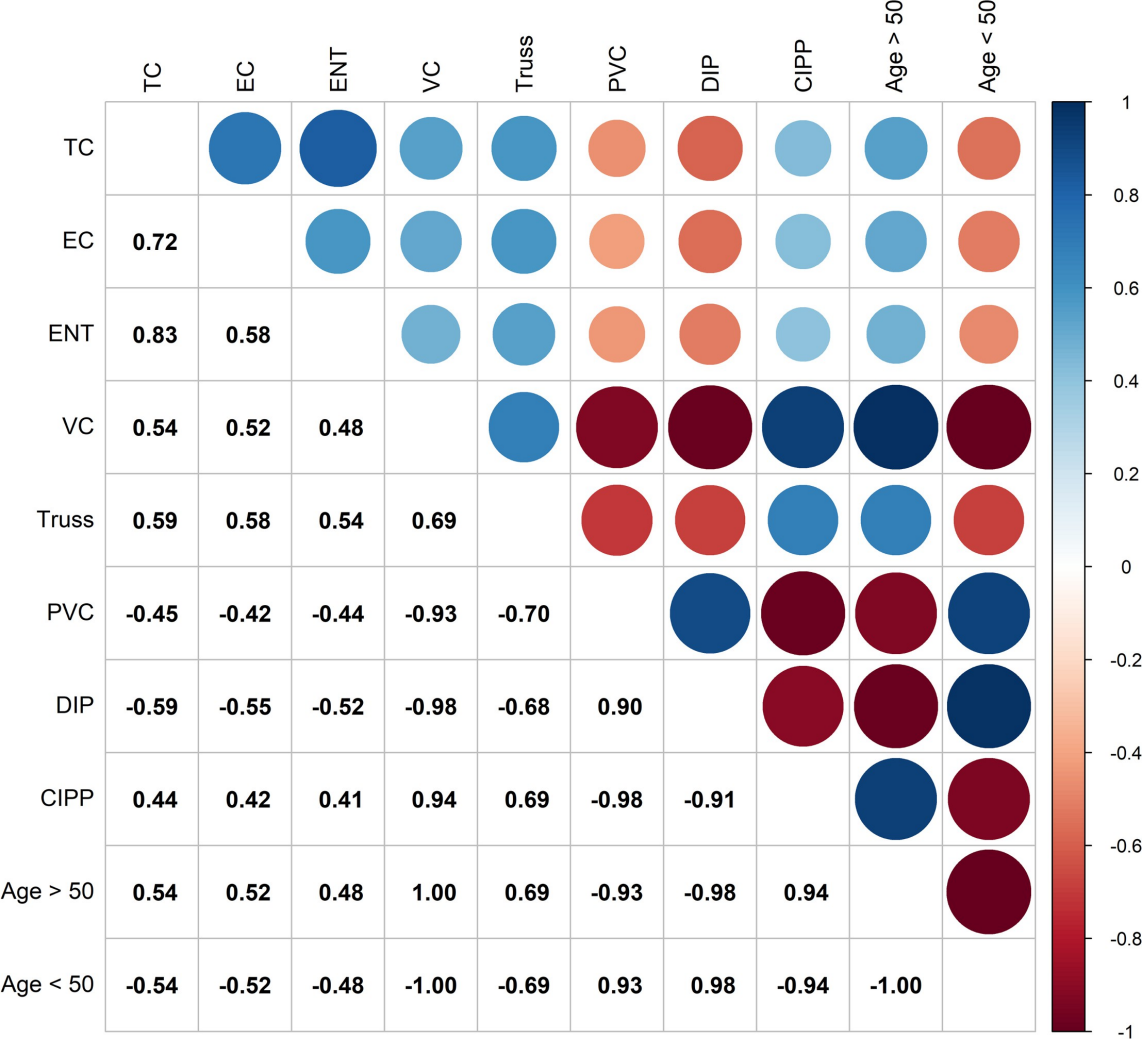
Correlation plot of Spearman rank correlations between molecular and infrastructure parameters. Significant (p < 0.05) correlations are shown on the upper plot as red dots (negative correlations) or blue dots (positive correlations) with the correlation strength represented by the shade of the dot. The size of the dot indicates the significance level with larger dots representing a higher probability that the identified correlation is real. The lower portion of the plot shows the spearman rank correlation values. TC = total coliforms, EC = *Escherichia coli*, ENT = *Enterococcus*, VC = vitrified clay, PVC = polyvinyl chloride, DIP = ductile iron pipe, CIPP = cured in place pipe, Age > 50 = percentage of pipe at each site greater than 50 years of age, Age <50 = percentage of pipe at each site less than 50 years of age.

The age of sewer pipes, pipe material, and proximity to stormwater discharge pipe outflows were also assessed and incorporated into our modelling effort. Within a 400-meter radius surrounding each site, between six to 76 pipes were observed. The percentage of pipes aged 50 years or older within a 400-meter radius of a sampling site were found to be significantly and positively correlated with FIB concentrations (ENT: r_s_ = 0.48, p < 0.001; EC: r_s_ = 0.52, p < 0.001) (Fig 3 and S7A and S7B Table). Sites within TCE headwaters (Sites 1, 2, and 3) were found to have the highest percentage of sewer pipes aged 50 years or older (Table 4). Additionally, pipes aged 50 years or older were found to be made of VC, which is known to be the least durable of the piping materials surrounding the estuary [4,46]. Multiple sampled sites are adjacent to stormwater outflows, but Sites 6 and 7 are the closest at five and 15 meters from the nearest outflow, respectively (Fig 1). Site 6 is located within a stormwater ditch finger of the estuary and Site 7 is downstream from Site 6. There are also two stormwater outflows between Sites 1 and 2, approximately 60–80 meters from each site, which may also influence the level of fecal contamination detected (Fig 1).

**Fig 4:**
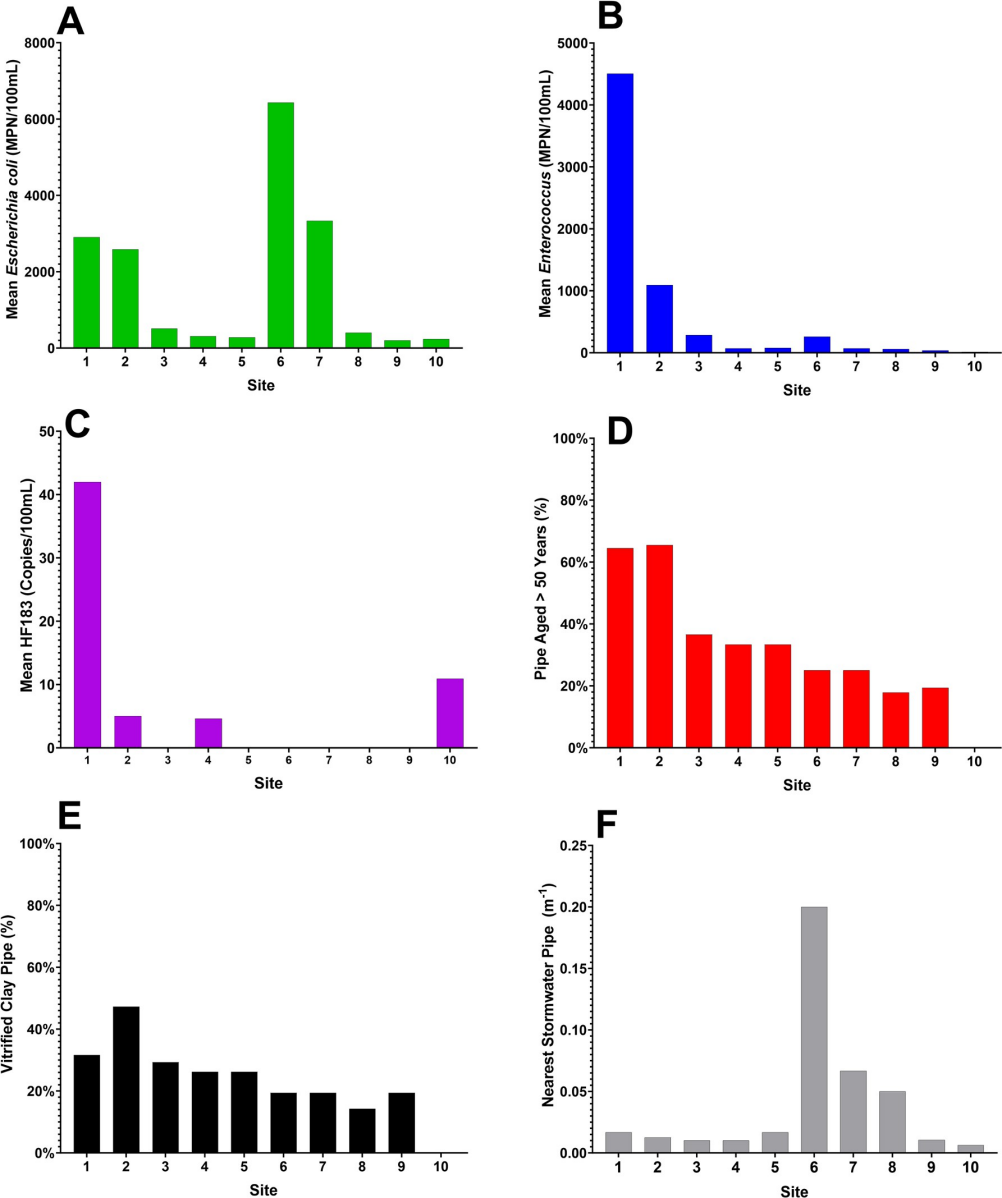
(A) Mean concentrations of *Escherichia coli* (EC) (MPN/100 mL), (B) *Enterococcus spp*. (ENT) (MPN/100 mL), (C) human host-specific marker HF183 (copies/100 mL), (D) percent of pipe aged over 50 years (Over50), (E) percentage of pipes constructed using vitrified clay (VC), and (F) the inverse of the nearest distance (m) to stormwater outflow pipes at each site presented as a stacked bar graph of the sum total at each site.

Sites with the highest mean EC, ENT, and HF183 concentrations frequently contained the largest percentage of sewer pipes aged over 50 and made of VC and/or were in close proximity to stormwater outflows (Fig 4 and S8A and S8B Table). The highest three ranked sites (Sites 1, 2, and 6) were the same across both ranking models and had elevated FIB concentrations. The remaining rankings varied, with sites containing elevated fecal contamination markers (such as Site 4) being ranked higher in the variable weighted model than the equal weighted model. In both ranking models, three of the top five sites ranked as potential sources of fecal contamination are within the upper estuary limits of TCE, suggesting a potential persistent source of contamination is located in this area.

## Discussion

Although estuarine water quality is routinely assessed using FIB and qMST approaches [47–51], municipalities struggle to use the data in the context of prioritizing infrastructure for monitoring and repair. Infrastructure remediation is a central priority of the Bipartisan Infrastructure Law [12], and collaborative conversations with city leaders in Beaufort, NC, emphasized the need to use a combination of water quality and infrastructure information to define locations for improvement. Here, we combined water quality data with GIS infrastructure data within a ranking system to prioritize sources of fecal contamination and locations for infrastructure remediation using TCE in Beaufort, NC, as a case study. We found that higher concentrations of FIB were identified in areas containing both aging sewer pipe, pipe materials prone to cracking, and stormwater outflows (Fig 4), suggesting local infrastructure may contribute to fecal contamination in the estuary. Additionally, local conditions, including weather events, contributed to levels of fecal contamination in the estuary.

Samples were collected over a range of wet and dry weather events. *Enterococcus spp*. concentrations were found to differ significantly between wet and dry weather whereas EC remained high in the estuary under both conditions. The human fecal marker, HF183, was detected infrequently but trended higher during wet weather conditions. However, DNA extraction recoveries were highly variable and total detections of this marker were low over the course of this study. Previous studies in TCE have detected similar levels of FIB and HF183 through large-scale sampling [1,2,10,18,19,27,34,47,51], and found significant increases in FIB concentrations following precipitation events [1,2,27,52]. Wet weather can increase the diffusion rate of fecal matter from soils into surrounding water bodies by infiltrating into and overwhelming compromised underground sewer infrastructure [28]. In fact, in a previous study examining inflow and infiltration across 19 wastewater treatment plants in the coastal areas of eastern NC, the Town of Beaufort was highlighted as being one of the systems most strongly impacted by rainfall and sea level rise [3]. Additionally, wet weather can increase the volume of stormwater runoff which can introduce fecal contaminants from urban areas into surrounding water bodies, especially in coastal communities where increased development replaces permeable surfaces [8,20,48]. Stormwater pipes are often found adjacent to sewer pipes and sewage can infiltrate into stormwater pipes through cracks and damaged areas of pipes [20,53]. In this study, the mean detection of EC was significantly higher at sites adjacent and downstream from stormwater outflows (Sites 6 and 7) and adjacent sewer infrastructure in the upper estuary (Sites 1 and 2) compared to other sampled locations (Fig 4).

Although precipitation events correlated with higher concentrations of FIB, persistent quantification of EC and ENT was also identified during dry weather events, indicating the potential for chronic sources of fecal contamination to be entering the TCE. Sites that had high levels fecal contamination observed during dry weather were located within TCE headwaters where tidal influences are minimal (Sites 1, 2, and 3). This conflicts with previous studies in the area, where dry weather FIB concentrations of this magnitude was not observed [18]. However, this previous study was completed more than a decade prior to the study presented here, and with the unprecedented rate of coastal development in the Town of Beaufort, the drivers of water quality impairment have changed. Other water quality monitoring studies have detected high levels of dry weather fecal contamination [8,20,53]. These studies suggests that the source of dry weather fecal contamination is most likely failing sewage infrastructure as sanitary sewage pipes are under constant stress due to human use which allows sewage to exfiltrate out of cracks in the pipes under all weather conditions [1,20,48,53]. These studies support our hypothesis that damaged or failing sewer infrastructure may contribute to the observed fecal contamination in the TCE.

Fecal indicator bacteria and HF183 were negatively correlated with salinity and dissolved oxygen concentrations in the estuary, and similar correlations have been found in other studies [10,54,55]. Korajkic and others [52] suggest the negative relationship between salinity and FIB and qMST markers is due to induced osmotic shock which affects the expression of genes associated with membrane composition therefore hindering survivability. Moreover, other water quality monitoring studies have also found a negative relationship between FIB species and dissolved oxygen [2,55]. Low dissolved oxygen can be caused by elevated levels of aerobic bacteria, like FIB, as they consume oxygen to perform metabolic processes. Thus, signs of lower dissolved oxygen are correlated with increased levels of bacteria.

No significant correlations were found to exist between FIB and environmental conditions including tide height, wind speed, and wind direction. This conflicts with a 2018 study by Kiaghadi and Rifai [56]: however, this difference may be due to the small size of the sampling area, and the general protective nature of the estuary in this study. TCE contained higher concentrations of EC and ENT in the upper TCE where tidal influences are lower and the residence time is longer compared to the downstream sites that had lower concentrations of EC and ENT and have stronger tidal influences and a shorter residence time. With the prevalence of King Tides increasing across eastern NC coastal systems, the influence of tidal inundation is already becoming more prevalent [10,57]. Further investigation is needed to fully ascertain the impact of these environmental conditions on fecal contamination levels across the TCE.

Significant and positive correlations were found between FIB concentrations and sewer pipe age and material. Sites with the highest rankings (Sites 1, 2, and 6) all have a high proportion of sewer pipes aged 50 years or older and are made of VC. Vitrified clay pipe material has been shown to be brittle and prone to failing [4,46]. The VC piping surrounding Sites 1, 2, and 6 was constructed in 1969 making these pipes approximately 52 years of age at the time of this study. In coastal areas where saltwater intrusion and storm events are frequent, VC pipes have a high potential of being compromised. Cracks and eroded areas allow sewage to exfiltrate into the estuary which could explain the high levels of fecal contamination observed in creek headwaters in TCE.

The ranking system defined in this study can be used in other systems to prioritize areas for infrastructure review and remediation. Here, we used two different weighting approaches to identify sites serving as potential sources of contamination. Although the top two “at-risk” sites were the same regardless of method, we suggest implementing the more rigorous variable weighting approach as this method uses empirical evidence of the included parameters which is more likely to capture sites serving as potential sources of contamination. This method places more emphasis on those sites with a measurable amount of contamination in the water while still allowing for other factors such as septic and sewage infrastructure around a site to help inform the final risk assessment.

This case study supports implementing a combined approach for infrastructure prioritization near priority waters. However, the present study raises additional questions which should be addressed in future research. More frequent sampling that corresponds to local weather conditions, including wet and dry weather events, will allow for the incorporation of climate in the ranking model. Sampling across tide heights is also recommended to assess the role of dilution in measured concentrations of indicator species. Future studies could also consider using additional host-associated markers to identify the source of fecal contamination, as a study in Southern California noted the value in using a variety of sewage indicators beyond HF183 to differentiate between sources when applying a ranking system to stormwater outflows [49]. Additional infrastructure information would be beneficial including flow, pipe diameter, and groundwater height as these factors can also contribute to fecal contamination; however, this information was not available in the Town of Beaufort records. Lastly, more sophisticated ranking models may be developed based on the case study presented here, and may include dye releases and/or tracking of other sewage-related chemical markers, such as caffeine and sucralose, to apply a more robust weighting method for parameters [50,58].

Ranking potential sources of fecal contamination may be essential to helping small coastal communities like the town of Beaufort, NC, to better allocate resources in a changing climate. The results of this study indicate that FIB concentrations were strongly associated with local infrastructure. By ranking the study sites based on surrounding infrastructure and average bacterial concentrations, several sites were determined to be most at risk for contributing fecal contamination to the estuary and should be prioritized for review and remediation. This method is broadly applicable to estuarine ecosystems and may help improve water quality through infrastructure repair.

## Conclusion

The simultaneous pressures of unprecedented rates of coastal development and aging and inundated sewage and stormwater infrastructure are forcing coastal towns across the coastal plains of southeastern USA to seek tools to prioritize infrastructure remediation. Given the increasing potential pressures on coastal waters, we strived to integrate routinely available microbial water quality monitoring, environmental parameter, and GIS-based infrastructure data into a framework that municipalities can use to prioritize sewage and stormwater infrastructure for potential remediation and to prevent contaminants from entering high priority recreational waters. Our study suggests that a simple ranking system can be used to integrate often readily available information which can then be applied to identify the magnitude and location of potential sources of fecal contamination in estuarine ecosystems, allowing municipalities to take action. In future applications of this ranking system, a long-term study conducted over 1–2 years may further elucidate the contribution of contamination from season, environmental, and infrastructure sources.

## Supporting information

S1 Fig Representative ddPCR fluorescence plots of A) HF183 positive control, and B) gyrA positive control.
(TIF)

S1 Table Water temperature, dissolved oxygen (DO), salinity, and turbidity values for all sites over the course of the project. NAs indicate sample data was unavailable for the site and date.
(DOCX)

S2 Table Concentration and lower/upper confidence intervals of total coliforms for each site and the method blank on each sampling date (MPN per 100mL). (DOCX)

S3 Table Concentration and lower/upper confidence intervals of *Escherichia coli* (*E. coli*) for each site and the method blank on each sampling date (MPN per100mL). (DOCX)

S4 Table Concentration and lower/upper confidence intervals of *Enterococcus* for each site and the method blank on each sampling date (MPN per100mL). NAs indicate sample data was unavailable for the site and date.
(DOCX)

S5 Table a. Spearman rank correlation test statistics (rs) between fecal indicator bacteria (FIB) species and with environmental conditions and parameters. b. Spearman rank correlation test p-values between fecal indicator bacteria (FIB) species with environmental conditions and parameters.
(DOCX)

S6 Table Concentrations of HF183 (copies per 100 mL) and extraction recovery percentage for all sites and the method blank over the course of the project. ND indicates sample concentration below the limit of detection for HF183.
(DOCX)

S7 Table a. Spearman rank correlation test statistics (rs) between fecal indicator bacteria (FIB) species with piping materials and construction dates. b. Spearman rank correlation test p-values between fecal indicator bacteria (FIB) species with piping materials and construction dates.
(DOCX)

S8 Table a. Rank results for each measured parameter using the equal weighting method. b. Rank results for each measured parameter using the variable weighting method.
(DOCX)

S9 Table Minimum information for publication of quantitative Real-Time PCR experiments guidelines checklist (MIQE). (XLSX)

S1 Method Design and sequence of the microbial source tracking control. (DOCX)

S2 Method Two parameter weighting methods for ranking potential sources of fecal contamination in Town Creek Estuary, Beaufort, North Carolina. (DOCX)

S1 Equation Equation to calculate gene copies/L from ddPCR. (DOCX)
