# Supplementary figures and images for: Using a combination of quantitative culture, molecular, and infrastructure data to rank potential sources of fecal contamination in Town Creek Estuary, North Carolina
Source: PLoS One. 2024 Apr 19;19(4):e0299254. doi: 10.1371/journal.pone.0299254 (PMC11029655; doi:10.1371/journal.pone.0299254)

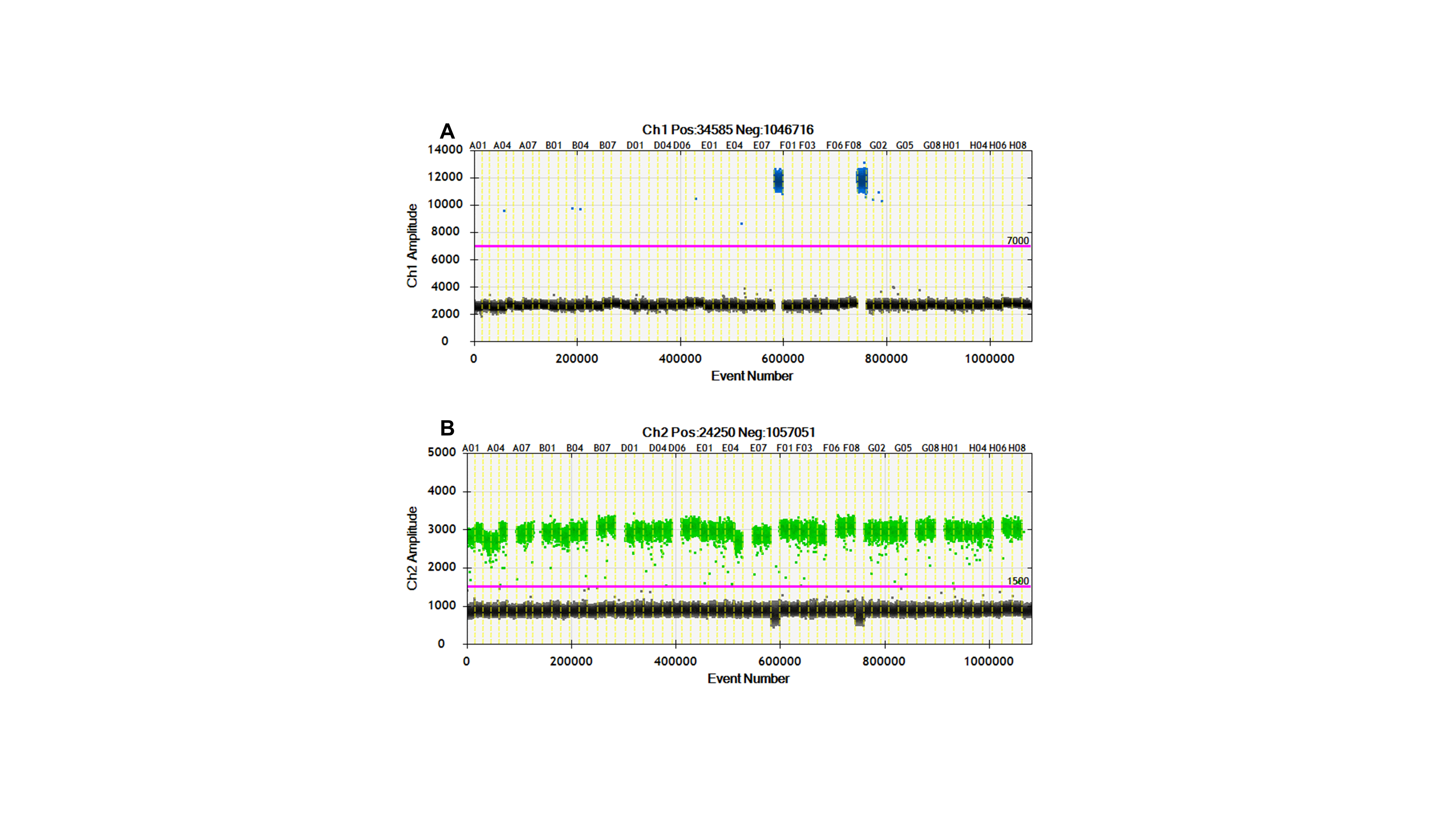

Supplement: S1 Fig — Representative ddPCR fluorescence plots of A) HF183 positive control, and B) gyrA positive control. (TIF) [file pone.0299254.s001.tif]
