# Supplementary material for: Using a combination of quantitative culture, molecular, and infrastructure data to rank potential sources of fecal contamination in Town Creek Estuary, North Carolina
Source: PLoS One. 2024 Apr 19;19(4):e0299254. doi: 10.1371/journal.pone.0299254 (PMC11029655; doi:10.1371/journal.pone.0299254)
Supplement: S1 Table — NAs indicate sample data was unavailable for the site and date. (DOCX) [file pone.0299254.s002.docx]

**S1 Table**. Water temperature, dissolved oxygen (DO), salinity, and turbidity values for all sites over the course of the project. NAs indicate sample data was unavailable for the site and date.

| Site | Site Description | Collection Date | Water  Temperature (˚C) | DO (%) | Salinity (ppt) | Turbidity (FNU) |
| --- | --- | --- | --- | --- | --- | --- |
| 1 | Ace Hardware | 8/6/2021 | 24.172 | 52.3 | 0.17 | 1.08 |
| 1 | Ace Hardware | 8/13/2021 | 26.704 | 45.9 | 0.46 | 5.54 |
| 1 | Ace Hardware | 8/27/2021 | 26.451 | 64.7 | 0.17 | 6.39 |
| 1 | Ace Hardware | 9/10/2021 | 24.323 | 38.4 | 18.64 | 7.52 |
| 1 | Ace Hardware | 9/20/2021 | NA | NA | NA | NA |
| 1 | Ace Hardware | 9/24/2021 | 23.082 | 41.5 | <0.1 | 2.42 |
| 1 | Ace Hardware | 9/28/2021 | 24.493 | 54.6 | 13.2 | 10.9 |
| 1 | Ace Hardware | 10/11/2021 | 22.349 | 5.30 | 2.41 | 5.83 |
| 2 | Channel-Ace Hardware | 8/6/2021 | 25.717 | 45.5 | 19.01 | 2.57 |
| 2 | Channel-Ace Hardware | 8/13/2021 | 31.848 | 80.2 | 28.01 | 3.80 |
| 2 | Channel-Ace Hardware | 8/27/2021 | 31.244 | 69.8 | 26.82 | 5.03 |
| 2 | Channel-Ace Hardware | 9/10/2021 | 27.035 | 87.9 | 34.95 | 4.54 |
| 2 | Channel-Ace Hardware | 9/20/2021 | NA | NA | NA | NA |
| 2 | Channel-Ace Hardware | 9/24/2021 | 24.528 | 81.5 | 34.1 | 4.93 |
| 2 | Channel-Ace Hardware | 9/28/2021 | 27.009 | 92 | 32.1 | 2.51 |
| 2 | Channel-Ace Hardware | 10/11/2021 | 23.313 | 6.43 | 27.29 | 4.47 |
| 3 | Stanton Road Finger | 8/6/2021 | 26.298 | 63.2 | 24.1 | 5.42 |
| 3 | Stanton Road Finger | 8/13/2021 | 31.522 | 100 | 30.09 | 2.95 |
| 3 | Stanton Road Finger | 8/27/2021 | 31.168 | 93.1 | 30.41 | 4.27 |
| 3 | Stanton Road Finger | 9/10/2021 | 26.975 | 93.3 | 35.19 | 3.64 |
| 3 | Stanton Road Finger | 9/20/2021 | NA | NA | NA | NA |
| 3 | Stanton Road Finger | 9/24/2021 | 24.786 | 92 | 35.3 | 4.51 |
| 3 | Stanton Road Finger | 9/28/2021 | 25.969 | 108 | 33.9 | 2.25 |
| 3 | Stanton Road Finger | 10/11/2021 | 23.272 | 6.66 | 29.17 | 4.27 |
| 4 | Stanton Road Channel | 8/6/2021 | 26.182 | 65.8 | 23.63 | 3.90 |
| 4 | Stanton Road Channel | 8/13/2021 | 31.69 | 98 | 30.32 | 3.17 |
| 4 | Stanton Road Channel | 8/27/2021 | 31.804 | 94.5 | 30.66 | 4.15 |
| 4 | Stanton Road Channel | 9/10/2021 | 27.28 | 94.2 | 35.12 | 3.06 |
| 4 | Stanton Road Channel | 9/20/2021 | NA | NA | NA | NA |
| 4 | Stanton Road Channel | 9/24/2021 | 24.872 | 90.4 | 35.2 | 2.89 |
| 4 | Stanton Road Channel | 9/28/2021 | 26.397 | 108.2 | 33.6 | 2.44 |
| 4 | Stanton Road Channel | 10/11/2021 | 23.309 | 6.69 | 29.18 | 4.52 |
| 5 | Marsh Finger | 8/6/2021 | 25.755 | 39.1 | 22.38 | 2.20 |
| 5 | Marsh Finger | 8/13/2021 | 32.676 | 96.4 | 29.75 | 1.67 |
| 5 | Marsh Finger | 8/27/2021 | 32.788 | 101.7 | 29.37 | 1.62 |
| 5 | Marsh Finger | 9/10/2021 | 27.297 | 98.2 | 35.2 | 2.64 |
| 5 | Marsh Finger | 9/20/2021 | NA | NA | NA | NA |
| 5 | Marsh Finger | 9/24/2021 | 24.258 | 86.1 | 34.3 | 29 |
| 5 | Marsh Finger | 9/28/2021 | 26.992 | 105 | 33.2 | 2.39 |
| 5 | Marsh Finger | 10/11/2021 | 23.349 | 6.68 | 28.27 | 3.89 |
| 6 | Stormwater Ditch Finger | 8/6/2021 | 26.119 | 59.9 | 22.91 | 2.63 |
| 6 | Stormwater Ditch Finger | 8/13/2021 | 31.448 | 101.7 | 29.63 | 3.45 |
| 6 | Stormwater Ditch Finger | 8/27/2021 | 31.663 | 93.3 | 27.71 | 7.23 |
| 6 | Stormwater Ditch Finger | 9/10/2021 | 26.711 | 96.8 | 35.17 | 4.11 |
| 6 | Stormwater Ditch Finger | 9/20/2021 | NA | NA | NA | NA |
| 6 | Stormwater Ditch Finger | 9/24/2021 | 24.741 | 89 | 34.9 | 328 |
| 6 | Stormwater Ditch Finger | 9/28/2021 | 26.413 | 112.4 | 33.6 | 2.38 |
| 6 | Stormwater Ditch Finger | 10/11/2021 | 23.295 | 6.38 | 30.52 | 5.31 |
| 7 | Channel Under Turner Street Bridge | 8/6/2021 | 26.168 | 62.8 | 23.75 | 4.10 |
| 7 | Channel Under Turner Street Bridge | 8/13/2021 | 31.471 | 94.6 | 30.36 | 3.15 |
| 7 | Channel Under Turner Street Bridge | 8/27/2021 | 31.422 | 90.1 | 30.55 | 5.4 |
| 7 | Channel Under Turner Street Bridge | 9/10/2021 | 26.962 | 91.3 | 35.2 | 3.47 |
| 7 | Channel Under Turner Street Bridge | 9/20/2021 | NA | NA | NA | NA |
| 7 | Channel Under Turner Street Bridge | 9/24/2021 | 25.01 | 92.8 | 35.2 | 4.28 |
| 7 | Channel Under Turner Street Bridge | 9/28/2021 | 26.02 | 104.8 | 33.9 | 2.81 |
| 7 | Channel Under Turner Street Bridge | 10/11/2021 | 23.328 | 6.86 | 29.53 | 4.20 |
| 8 | Town Creek Lift Station | 8/6/2021 | 26.393 | 66.6 | 24.4 | 5.12 |
| 8 | Town Creek Lift Station | 8/13/2021 | 31.586 | 102.7 | 29.95 | 5.93 |
| 8 | Town Creek Lift Station | 8/27/2021 | 31.467 | 92.4 | 28.82 | 6.14 |
| 8 | Town Creek Lift Station | 9/10/2021 | 27.504 | 106.8 | 35.34 | 14.99 |
| 8 | Town Creek Lift Station | 9/20/2021 | NA | NA | NA | NA |
| 8 | Town Creek Lift Station | 9/24/2021 | 25.465 | 87.1 | 35.4 | 10.03 |
| 8 | Town Creek Lift Station | 9/28/2021 | 27.052 | 111.1 | 33.2 | 3.26 |
| 8 | Town Creek Lift Station | 10/11/2021 | 23.170 | 6.61 | 31.69 | 6.27 |
| 9 | Public Access Dock | 8/6/2021 | 26.542 | 79.2 | 25.44 | 3.21 |
| 9 | Public Access Dock | 8/13/2021 | 31.458 | 101 | 30.63 | 3.11 |
| 9 | Public Access Dock | 8/27/2021 | 31.222 | 98.4 | 30.68 | 3.23 |
| 9 | Public Access Dock | 9/10/2021 | 26.618 | 95.7 | 35.39 | 6.54 |
| 9 | Public Access Dock | 9/20/2021 | NA | NA | NA | NA |
| 9 | Public Access Dock | 9/24/2021 | 25.161 | 92 | 25.161 | 4.76 |
| 9 | Public Access Dock | 9/28/2021 | 25.815 | 107 | 34.1 | 3.36 |
| 9 | Public Access Dock | 10/11/2021 | 23.153 | 6.69 | 31.84 | 6.21 |
| 10 | Town Creek Marina | 8/6/2021 | 26.049 | 81.1 | 24.54 | 2.09 |
| 10 | Town Creek Marina | 8/13/2021 | 31.146 | 98.7 | 31.49 | 2.68 |
| 10 | Town Creek Marina | 8/27/2021 | 30.7 | 95.8 | 31.92 | 3.6 |
| 10 | Town Creek Marina | 9/10/2021 | 26.614 | 97 | 35.94 | 3.49 |
| 10 | Town Creek Marina | 9/20/2021 | NA | NA | NA | NA |
| 10 | Town Creek Marina | 9/24/2021 | 24.969 | 93.7 | 36.4 | 4.83 |
| 10 | Town Creek Marina | 9/28/2021 | 25.246 | 102.7 | 34.7 | 1.91 |
| 10 | Town Creek Marina | 10/11/2021 | 23.129 | 6.43 | 30.48 | 4.45 |
