# Supplementary material for: Using a combination of quantitative culture, molecular, and infrastructure data to rank potential sources of fecal contamination in Town Creek Estuary, North Carolina
Source: PLoS One. 2024 Apr 19;19(4):e0299254. doi: 10.1371/journal.pone.0299254 (PMC11029655; doi:10.1371/journal.pone.0299254)
Supplement: S2 Table — (DOCX) [file pone.0299254.s003.docx]

**S2 Table**. Concentration and lower/upper confidence intervals of total coliforms for each site and the method blank on each sampling date (MPN per 100mL).

| Site | Site Description | Collection Date | Total Coliforms (MPN per 100mL) | Lower 95% Confidence Interval (MPN per 100 mL) | Upper 95% Confidence Interval (MPN per 100 mL) |
| --- | --- | --- | --- | --- | --- |
| 1 | Ace Hardware | 8/6/2021 | 24196 | 16304 | 47161 |
| 2 | Channel-Ace Hardware | 8/6/2021 | 24197 | NA | Infinite |
| 3 | Stanton Road Finger | 8/6/2021 | 22029.5 | 14262 | 40081.5 |
| 4 | Stanton Road Channel | 8/6/2021 | 24196.5 | 16304 | 47161 |
| 5 | Marsh Finger | 8/6/2021 | 15732.5 | 10463 | 24055.5 |
| 6 | Stormwater Ditch Finger | 8/6/2021 | 24197 | NA | Infinite |
| 7 | Channel Under Turner Street Bridge | 8/6/2021 | 24196 | 16304 | 47161 |
| 8 | Town Creek Lift Station | 8/6/2021 | 14264 | 9611.5 | 21617.5 |
| 9 | Public Access Dock | 8/6/2021 | 3441 | 2453 | 4725 |
| 10 | Town Creek Marina | 8/6/2021 | 2370.5 | 1621 | 3504 |
| 11 | Method Blank | 8/6/2021 | 9 | 0 | 37 |
| 1 | Ace Hardware | 8/13/2021 | 24197 | NA | Infinite |
| 2 | Channel-Ace Hardware | 8/13/2021 | 13084.5 | 8678.5 | 19261.5 |
| 3 | Stanton Road Finger | 8/13/2021 | 8239 | 5476 | 11654.5 |
| 4 | Stanton Road Channel | 8/13/2021 | 1906 | 1358.5 | 2611.5 |
| 5 | Marsh Finger | 8/13/2021 | 1808.5 | 1274 | 2509 |
| 6 | Stormwater Ditch Finger | 8/13/2021 | 6668 | 4442.5 | 9691 |
| 7 | Channel Under Turner Street Bridge | 8/13/2021 | 2121.5 | 1556.5 | 2847.5 |
| 8 | Town Creek Lift Station | 8/13/2021 | 6819.5 | 4541.5 | 9895.5 |
| 9 | Public Access Dock | 8/13/2021 | 818 | 599 | 1080 |
| 10 | Town Creek Marina | 8/13/2021 | 509 | 358 | 700.5 |
| 11 | Method Blank | 8/13/2021 | 9 | 0 | 37 |
| 1 | Ace Hardware | 8/27/2021 | 24197 | NA | Infinite |
| 2 | Channel-Ace Hardware | 8/27/2021 | 24196.5 | 16304 | 47161 |
| 3 | Stanton Road Finger | 8/27/2021 | 19864 | 10162 | 23531 |
| 4 | Stanton Road Channel | 8/27/2021 | 3136.5 | 2108.5 | 4478 |
| 5 | Marsh Finger | 8/27/2021 | 921 | 656.5 | 1259.5 |
| 6 | Stormwater Ditch Finger | 8/27/2021 | 24196 | 16304 | 47161 |
| 7 | Channel Under Turner Street Bridge | 8/27/2021 | 4621 | 2990 | 6839.5 |
| 8 | Town Creek Lift Station | 8/27/2021 | 9208 | 6205 | 12820 |
| 9 | Public Access Dock | 8/27/2021 | 1446.5 | 1026 | 1945 |
| 10 | Town Creek Marina | 8/27/2021 | 1932.5 | 1354.5 | 2703.5 |
| 11 | Method Blank | 8/27/2021 | 9 | 0 | 37 |
| 1 | Ace Hardware | 9/10/2021 | 24197 | NA | Infinite |
| 2 | Channel-Ace Hardware | 9/10/2021 | 10918.5 | 7393 | 15804.5 |
| 3 | Stanton Road Finger | 9/10/2021 | 1194.5 | 910 | 1540.5 |
| 4 | Stanton Road Channel | 9/10/2021 | 798.5 | 585 | 1063 |
| 5 | Marsh Finger | 9/10/2021 | 861.5 | 640 | 1127 |
| 6 | Stormwater Ditch Finger | 9/10/2021 | 4724 | 3055 | 7013.5 |
| 7 | Channel Under Turner Street Bridge | 9/10/2021 | 1118 | 819 | 1469 |
| 8 | Town Creek Lift Station | 9/10/2021 | 888 | 650.5 | 1176.5 |
| 9 | Public Access Dock | 9/10/2021 | 876 | 634 | 1168.5 |
| 10 | Town Creek Marina | 9/10/2021 | 683 | 511.5 | 928.5 |
| 11 | Method Blank | 9/10/2021 | 9 | 0 | 37 |
| 1 | Ace Hardware | 9/20/2021 | 24197 | NA | Infinite |
| 2 | Channel-Ace Hardware | 9/20/2021 | 14833.5 | 9705.5 | 22273.5 |
| 3 | Stanton Road Finger | 9/20/2021 | 5073 | 3276.5 | 7486 |
| 4 | Stanton Road Channel | 9/20/2021 | 4726.5 | 3223 | 6702 |
| 5 | Marsh Finger | 9/20/2021 | 2617.5 | 1856 | 16247.5 |
| 6 | Stormwater Ditch Finger | 9/20/2021 | 5361 | 3661.5 | 7523 |
| 7 | Channel Under Turner Street Bridge | 9/20/2021 | 3937.5 | 2670 | 5665.5 |
| 8 | Town Creek Lift Station | 9/20/2021 | 3289 | 2179.5 | 4801 |
| 9 | Public Access Dock | 9/20/2021 | 1552 | 1150.5 | 2049.5 |
| 10 | Town Creek Marina | 9/20/2021 | 1275.5 | 945.5 | 1673 |
| 11 | Method Blank | 9/20/2021 | 9 | 0 | 37 |
| 1 | Ace Hardware | 9/24/2021 | 24196.5 | 16304 | 47161 |
| 2 | Channel-Ace Hardware | 9/24/2021 | 24197 | NA | Infinite |
| 3 | Stanton Road Finger | 9/24/2021 | 6019.5 | 3938.5 | 8690 |
| 4 | Stanton Road Channel | 9/24/2021 | 4390.5 | 2978.5 | 6311.5 |
| 5 | Marsh Finger | 9/24/2021 | 10501.5 | 7076 | 15121 |
| 6 | Stormwater Ditch Finger | 9/24/2021 | 24197 | NA | Infinite |
| 7 | Channel Under Turner Street Bridge | 9/24/2021 | 24197 | NA | Infinite |
| 8 | Town Creek Lift Station | 9/24/2021 | 7229 | 4814.5 | 10463 |
| 9 | Public Access Dock | 9/24/2021 | 2965 | 2089.5 | 4113.5 |
| 10 | Town Creek Marina | 9/24/2021 | 1924.5 | 1354.5 | 2668 |
| 11 | Method Blank | 9/24/2021 | 9 | 0 | 37 |
| 1 | Ace Hardware | 9/28/2021 | 24197 | NA | Infinite |
| 2 | Channel-Ace Hardware | 9/28/2021 | 4522.5 | 2921.5 | 6653 |
| 3 | Stanton Road Finger | 9/28/2021 | 7046 | 4652.5 | 10017.5 |
| 4 | Stanton Road Channel | 9/28/2021 | 2251 | 1496 | 3404.5 |
| 5 | Marsh Finger | 9/28/2021 | 1377 | 968.5 | 1918.5 |
| 6 | Stormwater Ditch Finger | 9/28/2021 | 2431.5 | 1637.5 | 3563.5 |
| 7 | Channel Under Turner Street Bridge | 9/28/2021 | 1791 | 1260.5 | 2492.5 |
| 8 | Town Creek Lift Station | 9/28/2021 | 2094.5 | 1390.5 | 3139 |
| 9 | Public Access Dock | 9/28/2021 | 794 | 566 | 1078.5 |
| 10 | Town Creek Marina | 9/28/2021 | 1244.5 | 887 | 1694.5 |
| 11 | Method Blank | 9/28/2021 | 9 | 0 | 37 |
| 1 | Ace Hardware | 10/11/2021 | 24197 | NA | Infinite |
| 2 | Channel-Ace Hardware | 10/11/2021 | 24197 | NA | Infinite |
| 3 | Stanton Road Finger | 10/11/2021 | 11729.5 | 7777 | 18528 |
| 4 | Stanton Road Channel | 10/11/2021 | 6221 | 4070.5 | 9062.5 |
| 5 | Marsh Finger | 10/11/2021 | 3827.5 | 2548.5 | 5448.5 |
| 6 | Stormwater Ditch Finger | 10/11/2021 | 24197 | NA | Infinite |
| 7 | Channel Under Turner Street Bridge | 10/11/2021 | 8454.5 | 5847.5 | 11880 |
| 8 | Town Creek Lift Station | 10/11/2021 | 5940.5 | 3842 | 8684 |
| 9 | Public Access Dock | 10/11/2021 | 6867 | 4493 | 9744 |
| 10 | Town Creek Marina | 10/11/2021 | 3454.5 | 2192.5 | 5268 |
| 11 | Method Blank | 10/11/2021 | 9 | 0 | 37 |
