# Supplementary material for: Using a combination of quantitative culture, molecular, and infrastructure data to rank potential sources of fecal contamination in Town Creek Estuary, North Carolina
Source: PLoS One. 2024 Apr 19;19(4):e0299254. doi: 10.1371/journal.pone.0299254 (PMC11029655; doi:10.1371/journal.pone.0299254)
Supplement: S3 Table — (DOCX) [file pone.0299254.s004.docx]

**S3 Table**. Concentration and lower/upper confidence intervals of *Escherichia coli* (*E. coli*) for each site and the method blank on each sampling date (MPN per 100mL).

| Site | Site Description | Collection Date | *E. coli* (MPN per 100mL) | | Lower 95% Confidence Interval (MPN per 100 mL) | Upper 95% Confidence Interval (MPN per 100 mL) |
| --- | --- | --- | --- | --- | --- | --- |
| 1 | Ace Hardware | 8/6/2021 | 381.5 | 257 | | 547 |
| 2 | Channel-Ace Hardware | 8/6/2021 | 1774 | 1230 | | 2529.5 |
| 3 | Stanton Road Finger | 8/6/2021 | 1275.5 | 904 | | 1787 |
| 4 | Stanton Road Channel | 8/6/2021 | 488 | 344 | | 669 |
| 5 | Marsh Finger | 8/6/2021 | 623 | 435.5 | | 863 |
| 6 | Stormwater Ditch Finger | 8/6/2021 | 187 | 110 | | 298 |
| 7 | Channel Under Turner Street Bridge | 8/6/2021 | 267.5 | 170 | | 394.5 |
| 8 | Town Creek Lift Station | 8/6/2021 | 240.5 | 151 | | 360 |
| 9 | Public Access Dock | 8/6/2021 | 97.5 | 48.5 | | 177.5 |
| 10 | Town Creek Marina | 8/6/2021 | 86.5 | 41.5 | | 155.5 |
| 11 | Method Blank | 8/6/2021 | 9 | 0 | | 37 |
| 1 | Ace Hardware | 8/13/2021 | 2861.5 | 1889 | | 4116.5 |
| 2 | Channel-Ace Hardware | 8/13/2021 | 1315.5 | 976.5 | | 1745 |
| 3 | Stanton Road Finger | 8/13/2021 | 758 | 535 | | 1034.5 |
| 4 | Stanton Road Channel | 8/13/2021 | 298.5 | 196 | | 435 |
| 5 | Marsh Finger | 8/13/2021 | 188.5 | 111 | | 297 |
| 6 | Stormwater Ditch Finger | 8/13/2021 | 946 | 683.5 | | 1271.5 |
| 7 | Channel Under Turner Street Bridge | 8/13/2021 | 211.5 | 128 | | 325 |
| 8 | Town Creek Lift Station | 8/13/2021 | 276 | 178.5 | | 409.5 |
| 9 | Public Access Dock | 8/13/2021 | 122 | 64.5 | | 215 |
| 10 | Town Creek Marina | 8/13/2021 | 148 | 83.5 | | 236.5 |
| 11 | Method Blank | 8/13/2021 | 9 | 0 | | 37 |
| 1 | Ace Hardware | 8/27/2021 | 1624.5 | 1175.5 | | 2221 |
| 2 | Channel-Ace Hardware | 8/27/2021 | 2056.5 | 1374.5 | | 3051.5 |
| 3 | Stanton Road Finger | 8/27/2021 | 610 | 435 | | 821.5 |
| 4 | Stanton Road Channel | 8/27/2021 | 196 | 115 | | 306 |
| 5 | Marsh Finger | 8/27/2021 | 142.5 | 79 | | 230.5 |
| 6 | Stormwater Ditch Finger | 8/27/2021 | 357.5 | 244.5 | | 505 |
| 7 | Channel Under Turner Street Bridge | 8/27/2021 | 290 | 187.5 | | 424.5 |
| 8 | Town Creek Lift Station | 8/27/2021 | 1019.5 | 737.5 | | 1371 |
| 9 | Public Access Dock | 8/27/2021 | 206.5 | 125 | | 321 |
| 10 | Town Creek Marina | 8/27/2021 | 217 | 134.5 | | 329.5 |
| 11 | Method Blank | 8/27/2021 | 9 | 0 | | 37 |
| 1 | Ace Hardware | 9/10/2021 | 6330.5 | 4142 | | 9108 |
| 2 | Channel-Ace Hardware | 9/10/2021 | 1392 | 1005.5 | | 1877.5 |
| 3 | Stanton Road Finger | 9/10/2021 | 285.5 | 184.5 | | 420 |
| 4 | Stanton Road Channel | 9/10/2021 | 131.5 | 70.5 | | 222.5 |
| 5 | Marsh Finger | 9/10/2021 | 312 | 201 | | 455 |
| 6 | Stormwater Ditch Finger | 9/10/2021 | 811 | 594 | | 1074.5 |
| 7 | Channel Under Turner Street Bridge | 9/10/2021 | 410 | 280.5 | | 582.5 |
| 8 | Town Creek Lift Station | 9/10/2021 | 176.5 | 100.5 | | 279.5 |
| 9 | Public Access Dock | 9/10/2021 | 226 | 137 | | 345.5 |
| 10 | Town Creek Marina | 9/10/2021 | 459 | 323 | | 630.5 |
| 11 | Method Blank | 9/10/2021 | 9 | 0 | | 37 |
| 1 | Ace Hardware | 9/20/2021 | 3454.5 | 2192.5 | | 5268 |
| 2 | Channel-Ace Hardware | 9/20/2021 | 8128 | 5420.5 | | 11781 |
| 3 | Stanton Road Finger | 9/20/2021 | 122 | 63 | | 206.5 |
| 4 | Stanton Road Channel | 9/20/2021 | 393 | 274 | | 539 |
| 5 | Marsh Finger | 9/20/2021 | 235 | 152 | | 349.5 |
| 6 | Stormwater Ditch Finger | 9/20/2021 | 410 | 277 | | 578 |
| 7 | Channel Under Turner Street Bridge | 9/20/2021 | 292 | 192 | | 422.5 |
| 8 | Town Creek Lift Station | 9/20/2021 | 364 | 245.5 | | 516.5 |
| 9 | Public Access Dock | 9/20/2021 | 262.5 | 169.5 | | 386.5 |
| 10 | Town Creek Marina | 9/20/2021 | 285.5 | 181.5 | | 420 |
| 11 | Method Blank | 9/20/2021 | 9 | 0 | | 37 |
| 1 | Ace Hardware | 9/24/2021 | 1422.5 | 998 | | 2003.5 |
| 2 | Channel-Ace Hardware | 9/24/2021 | 3900 | 2475.5 | | 5853.5 |
| 3 | Stanton Road Finger | 9/24/2021 | 222 | 136.5 | | 339 |
| 4 | Stanton Road Channel | 9/24/2021 | 334 | 222 | | 483.5 |
| 5 | Marsh Finger | 9/24/2021 | 288 | 186 | | 427 |
| 6 | Stormwater Ditch Finger | 9/24/2021 | 24196.5 | 16304 | | 47161 |
| 7 | Channel Under Turner Street Bridge | 9/24/2021 | 24196.5 | 16304 | | 47161 |
| 8 | Town Creek Lift Station | 9/24/2021 | 176 | 103.5 | | 276 |
| 9 | Public Access Dock | 9/24/2021 | 77.5 | 32.5 | | 146 |
| 10 | Town Creek Marina | 9/24/2021 | 169.5 | 96 | | 270.5 |
| 11 | Method Blank | 9/24/2021 | 9 | 0 | | 37 |
| 1 | Ace Hardware | 9/28/2021 | 1542.5 | 1095 | | 2161 |
| 2 | Channel-Ace Hardware | 9/28/2021 | 463 | 326 | | 636.5 |
| 3 | Stanton Road Finger | 9/28/2021 | 421.5 | 286 | | 590 |
| 4 | Stanton Road Channel | 9/28/2021 | 313.5 | 204.5 | | 457.5 |
| 5 | Marsh Finger | 9/28/2021 | 172.5 | 100 | | 274.5 |
| 6 | Stormwater Ditch Finger | 9/28/2021 | 332.5 | 219 | | 478.5 |
| 7 | Channel Under Turner Street Bridge | 9/28/2021 | 283.5 | 180 | | 421.5 |
| 8 | Town Creek Lift Station | 9/28/2021 | 282.5 | 182.5 | | 410.5 |
| 9 | Public Access Dock | 9/28/2021 | 90 | 41.5 | | 162.5 |
| 10 | Town Creek Marina | 9/28/2021 | 128.5 | 72 | | 221.5 |
| 11 | Method Blank | 9/28/2021 | 9 | 0 | | 37 |
| 1 | Ace Hardware | 10/11/2021 | 5643.5 | 3907 | | 7908.5 |
| 2 | Channel-Ace Hardware | 10/11/2021 | 1682.5 | 1199.5 | | 2320.5 |
| 3 | Stanton Road Finger | 10/11/2021 | 412.5 | 283 | | 586.5 |
| 4 | Stanton Road Channel | 10/11/2021 | 313 | 205.5 | | 453 |
| 5 | Marsh Finger | 10/11/2021 | 297.5 | 192 | | 439.5 |
| 6 | Stormwater Ditch Finger | 10/11/2021 | 24196.5 | 16304 | | 47161 |
| 7 | Channel Under Turner Street Bridge | 10/11/2021 | 697 | 492 | | 959.5 |
| 8 | Town Creek Lift Station | 10/11/2021 | 698.5 | 502 | | 945 |
| 9 | Public Access Dock | 10/11/2021 | 521 | 363 | | 720 |
| 10 | Town Creek Marina | 10/11/2021 | 388 | 265.5 | | 549.5 |
| 11 | Method Blank | 10/11/2021 | 9 | 0 | | 37 |
