# Supplementary material for: Using a combination of quantitative culture, molecular, and infrastructure data to rank potential sources of fecal contamination in Town Creek Estuary, North Carolina
Source: PLoS One. 2024 Apr 19;19(4):e0299254. doi: 10.1371/journal.pone.0299254 (PMC11029655; doi:10.1371/journal.pone.0299254)
Supplement: S4 Table — NAs indicate sample data was unavailable for the site and date. (DOCX) [file pone.0299254.s005.docx]

**S4 Table**. Concentration and lower/upper confidence intervals of *Enterococcus* for each site and the method blank on each sampling date (MPN per 100mL). NAs indicate sample data was unavailable for the site and date.

| Site | Site Description | Collection Date | *Enterococcus* spp. (MPN per 100mL) | Lower 95% Confidence Interval (MPN per 100 mL) | Upper 95% Confidence Interval (MPN per 100 mL) |
| --- | --- | --- | --- | --- | --- |
| 1 | Ace Hardware | 8/6/2021 | 443.5 | 303.5 | 619.5 |
| 2 | Channel-Ace Hardware | 8/6/2021 | 1320.5 | 956.5 | 1789.5 |
| 3 | Stanton Road Finger | 8/6/2021 | 1485 | 1072 | 1988 |
| 4 | Stanton Road Channel | 8/6/2021 | 140 | 78 | 235 |
| 5 | Marsh Finger | 8/6/2021 | 30.5 | 9 | 72.5 |
| 6 | Stormwater Ditch Finger | 8/6/2021 | 894.5 | 656 | 1199.5 |
| 7 | Channel Under Turner Street Bridge | 8/6/2021 | 187 | 108 | 300 |
| 8 | Town Creek Lift Station | 8/6/2021 | 25.5 | 9 | 75 |
| 9 | Public Access Dock | 8/6/2021 | 9 | 0 | 37 |
| 10 | Town Creek Marina | 8/6/2021 | 14.5 | 1.5 | 54 |
| 11 | Method Blank | 8/6/2021 | 9 | 0 | 37 |
| 1 | Ace Hardware | 8/13/2021 | 260.5 | 162.5 | 392.5 |
| 2 | Channel-Ace Hardware | 8/13/2021 | 15 | 2 | 63 |
| 3 | Stanton Road Finger | 8/13/2021 | 9.5 | 0.5 | 46 |
| 4 | Stanton Road Channel | 8/13/2021 | 9.5 | 0.5 | 46 |
| 5 | Marsh Finger | 8/13/2021 | 9.5 | 0.5 | 46 |
| 6 | Stormwater Ditch Finger | 8/13/2021 | 31 | 7 | 89 |
| 7 | Channel Under Turner Street Bridge | 8/13/2021 | 9.5 | 0.5 | 46 |
| 8 | Town Creek Lift Station | 8/13/2021 | 52 | 23 | 116 |
| 9 | Public Access Dock | 8/13/2021 | 9 | 0 | 37 |
| 10 | Town Creek Marina | 8/13/2021 | 9.5 | 0.5 | 46 |
| 11 | Method Blank | 8/13/2021 | 9 | 0 | 37 |
| 1 | Ace Hardware | 8/27/2021 | 512.5 | 355.5 | 715 |
| 2 | Channel-Ace Hardware | 8/27/2021 | 86 | 40.5 | 161 |
| 3 | Stanton Road Finger | 8/27/2021 | 9 | 0 | 37 |
| 4 | Stanton Road Channel | 8/27/2021 | 20 | 3.5 | 63 |
| 5 | Marsh Finger | 8/27/2021 | 9 | 0 | 37 |
| 6 | Stormwater Ditch Finger | 8/27/2021 | 52 | 21 | 111 |
| 7 | Channel Under Turner Street Bridge | 8/27/2021 | 10 | 1 | 55 |
| 8 | Town Creek Lift Station | 8/27/2021 | 30.5 | 7.5 | 81 |
| 9 | Public Access Dock | 8/27/2021 | 9 | 0 | 37 |
| 10 | Town Creek Marina | 8/27/2021 | 9 | 0 | 37 |
| 11 | Method Blank | 8/27/2021 | 9 | 0 | 37 |
| 1 | Ace Hardware | 9/10/2021 | 3591 | 2279.5 | 5450.5 |
| 2 | Channel-Ace Hardware | 9/10/2021 | 41.5 | 12.5 | 98.5 |
| 3 | Stanton Road Finger | 9/10/2021 | 9.5 | 0.5 | 46 |
| 4 | Stanton Road Channel | 9/10/2021 | 9.5 | 0.5 | 46 |
| 5 | Marsh Finger | 9/10/2021 | 9 | 0 | 37 |
| 6 | Stormwater Ditch Finger | 9/10/2021 | 20.5 | 4 | 72 |
| 7 | Channel Under Turner Street Bridge | 9/10/2021 | 9 | 0 | 37 |
| 8 | Town Creek Lift Station | 9/10/2021 | 14.5 | 1.5 | 54 |
| 9 | Public Access Dock | 9/10/2021 | 9.5 | 0.5 | 46 |
| 10 | Town Creek Marina | 9/10/2021 | 9 | 0 | 37 |
| 11 | Method Blank | 9/10/2021 | 9 | 0 | 37 |
| 1 | Ace Hardware | 9/20/2021 | 1596.5 | 1137 | 2201 |
| 2 | Channel-Ace Hardware | 9/20/2021 | 122.5 | 67.5 | 217.5 |
| 3 | Stanton Road Finger | 9/20/2021 | 20.5 | 4 | 72 |
| 4 | Stanton Road Channel | 9/20/2021 | 20 | 3 | 71 |
| 5 | Marsh Finger | 9/20/2021 | 20 | 3.5 | 63 |
| 6 | Stormwater Ditch Finger | 9/20/2021 | 115 | 61 | 202.5 |
| 7 | Channel Under Turner Street Bridge | 9/20/2021 | 36 | 12 | 92 |
| 8 | Town Creek Lift Station | 9/20/2021 | 15 | 2 | 57 |
| 9 | Public Access Dock | 9/20/2021 | 14.5 | 1.5 | 54 |
| 10 | Town Creek Marina | 9/20/2021 | 9 | 0 | 37 |
| 11 | Method Blank | 9/20/2021 | 9 | 0 | 37 |
| 1 | Ace Hardware | 9/24/2021 | 929.5 | 662.5 | 1267.5 |
| 2 | Channel-Ace Hardware | 9/24/2021 | 4957.5 | 3318 | 7027 |
| 3 | Stanton Road Finger | 9/24/2021 | 121.5 | 65 | 209 |
| 4 | Stanton Road Channel | 9/24/2021 | 135 | 76.5 | 232.5 |
| 5 | Marsh Finger | 9/24/2021 | 255.5 | 163 | 380 |
| 6 | Stormwater Ditch Finger | 9/24/2021 | 239 | 148 | 367 |
| 7 | Channel Under Turner Street Bridge | 9/24/2021 | 63 | 29 | 137 |
| 8 | Town Creek Lift Station | 9/24/2021 | 81 | 41 | 153 |
| 9 | Public Access Dock | 9/24/2021 | 52.5 | 19.5 | 116.5 |
| 10 | Town Creek Marina | 9/24/2021 | 14.5 | 1.5 | 54 |
| 11 | Method Blank | 9/24/2021 | 9 | 0 | 37 |
| 1 | Ace Hardware | 9/28/2021 | NA | NA | NA |
| 2 | Channel-Ace Hardware | 9/28/2021 | NA | NA | NA |
| 3 | Stanton Road Finger | 9/28/2021 | NA | NA | NA |
| 4 | Stanton Road Channel | 9/28/2021 | NA | NA | NA |
| 5 | Marsh Finger | 9/28/2021 | NA | NA | NA |
| 6 | Stormwater Ditch Finger | 9/28/2021 | NA | NA | NA |
| 7 | Channel Under Turner Street Bridge | 9/28/2021 | NA | NA | NA |
| 8 | Town Creek Lift Station | 9/28/2021 | NA | NA | NA |
| 9 | Public Access Dock | 9/28/2021 | NA | NA | NA |
| 10 | Town Creek Marina | 9/28/2021 | NA | NA | NA |
| 11 | Method Blank | 9/28/2021 | NA | NA | NA |
| 1 | Ace Hardware | 10/11/2021 | 24196.5 | 16304 | 47161 |
| 2 | Channel-Ace Hardware | 10/11/2021 | 1114 | 806 | 1507.5 |
| 3 | Stanton Road Finger | 10/11/2021 | 338 | 225 | 491 |
| 4 | Stanton Road Channel | 10/11/2021 | 166.5 | 97.5 | 273 |
| 5 | Marsh Finger | 10/11/2021 | 208.5 | 126.5 | 327.5 |
| 6 | Stormwater Ditch Finger | 10/11/2021 | 442.5 | 302.5 | 619 |
| 7 | Channel Under Turner Street Bridge | 10/11/2021 | 171.5 | 97.5 | 276.5 |
| 8 | Town Creek Lift Station | 10/11/2021 | 186 | 109 | 296 |
| 9 | Public Access Dock | 10/11/2021 | 158.5 | 88 | 254.5 |
| 10 | Town Creek Marina | 10/11/2021 | 20.5 | 4 | 72 |
| 11 | Method Blank | 10/11/2021 | 9 | 0 | 37 |
