# Supplementary material for: Using a combination of quantitative culture, molecular, and infrastructure data to rank potential sources of fecal contamination in Town Creek Estuary, North Carolina
Source: PLoS One. 2024 Apr 19;19(4):e0299254. doi: 10.1371/journal.pone.0299254 (PMC11029655; doi:10.1371/journal.pone.0299254)
Supplement: S5 Table — a. Spearman rank correlation test statistics (rs) between fecal indicator bacteria (FIB) species and with environmental conditions and parameters. b. Spearman rank correlation test p-values between fecal indicator bacteria (FIB) species with environmental conditions and parameters. (DOCX) [file pone.0299254.s006.docx]

**S5a Table**. Spearman rank correlation test statistics (r_s_) between fecal indicator bacteria (FIB) species with environmental conditions and parameters. TC=total coliforms, EC=*Escherichia coli*, ENT=*Enterococcus*

|  | TC | EC | ENT | 24-Hr Rainfall | 72-Hr Rainfall | Tide Height | Wind Speed | Wind Direction | Water Temperature | Dissolved Oxygen | Salinity | Turbidity |
| --- | --- | --- | --- | --- | --- | --- | --- | --- | --- | --- | --- | --- |
| TC | 1 | 0.73*** | 0.81*** | 0.23* | 0.44*** | -0.15 | -0.03 | -0.15 | -0.42*** | -0.64*** | -0.57*** | 0.33*** |
| EC | 0.73*** | 1 | 0.57*** | 0.15 | 0.08 | 0.22*** | 0.17 | 0.11 | -0.32* | -0.47*** | -0.25* | 0.31** |
| ENT | 0.81*** | 0.57*** | 1 | 0.5*** | 0.52*** | 0.14 | 0.24 | 0.09 | -0.68*** | -0.74*** | -0.48*** | 0.37** |
| 24-Hr Rainfall | 0.23* | 0.15 | 0.5*** | 1 | 0.56*** | 0.53*** | 0.74*** | 0.65*** | -0.86*** | -0.5*** | 0.22 | 0.25** |
| 72-Hr Rainfall | 0.44*** | 0.08 | 0.52*** | 0.56*** | 1 | -0.14 | -0.09 | -0.26 | -0.69*** | -0.72*** | -0.38** | 0.03 |
| Tide Height | -0.15 | 0.22* | 0.14 | 0.53*** | -0.14 | 1 | 0.83*** | 0.77*** | -0.48** | -0.21* | 0.45** | 0.31** |
| Wind Speed | -0.03 | 0.17 | 0.24 | 0.74*** | -0.09 | 0.83*** | 1 | 0.94*** | -0.53** | -0.13 | 0.51*** | 0.34*** |
| Wind Direction | -0.15 | 0.11 | 0.09 | 0.65*** | -0.26 | 0.77*** | 0.94*** | 1 | -0.37*** | 0.08 | 0.63*** | 0.31* |
| Water Temperature | -0.42*** | -0.32* | -0.68*** | -0.86*** | -0.69*** | -0.48** | -0.53** | -0.37*** | 1 | 0.78*** | 0.12 | -0.24 |
| Dissolved Oxygen | -0.64*** | -0.47*** | -0.74*** | -0.5*** | -0.72*** | -0.21* | -0.13 | 0.08 | 0.78*** | 1 | 0.51*** | -0.22** |
| Salinity | -0.57*** | -0.25* | -0.48*** | 0.22 | -0.38** | 0.45** | 0.51*** | 0.63*** | 0.12 | 0.51*** | 1 | 0.15 |
| Turbidity | 0.33*** | 0.31** | 0.37** | 0.25** | 0.03 | 0.31** | 0.34*** | 0.31* | -0.24 | -0.22** | 0.15 | 1 |

(* = p < 0.05, ** = p < 0.01, and *** = p < 0.001)

**S5b Table**. Spearman rank correlation test p-values between fecal indicator bacteria (FIB) species with environmental conditions and parameters. TC=total coliforms, EC= *Escherichia coli*, ENT=*Enterococcus*

|  | TC | EC | ENT | 24-Hr Rainfall | 72-Hr Rainfall | Tide Height | Wind Speed | Wind Direction | Water Temperature | Dissolved Oxygen | Salinity | Turbidity |
| --- | --- | --- | --- | --- | --- | --- | --- | --- | --- | --- | --- | --- |
| TC | 0.00E+00 | 9.60E-15 | 1.46E-18 | 1.33E-02 | 2.30E-05 | 6.12E-01 | 6.03E-01 | 3.21E-01 | 5.09E-04 | 1.86E-10 | 1.78E-07 | 4.71E-04 |
| EC | 9.60E-15 | 0.00E+00 | 1.34E-07 | 1.55E-01 | 2.30E-01 | 4.79E-02 | 1.66E-01 | 3.47E-01 | 1.22E-02 | 1.48E-05 | 2.20E-02 | 3.12E-03 |
| ENT | 1.46E-18 | 1.34E-07 | 0.00E+00 | 6.75E-05 | 1.60E-06 | 1.75E-01 | 7.40E-02 | 6.24E-01 | 2.40E-09 | 2.20E-11 | 1.04E-04 | 3.25E-03 |
| 24-Hr Rainfall | 1.33E-02 | 1.55E-01 | 6.75E-05 | 0.00E+00 | 4.28E-11 | 2.41E-07 | 3.87E-13 | 1.02E-08 | 3.74E-14 | 1.86E-07 | 2.47E-01 | 5.18E-03 |
| 72-Hr Rainfall | 2.30E-05 | 2.30E-01 | 1.60E-06 | 4.28E-11 | 0.00E+00 | 4.25E-01 | 6.73E-01 | 1.11E-01 | 2.65E-07 | 2.31E-13 | 2.15E-03 | 9.05E-02 |
| Tide Height | 6.12E-01 | 4.79E-02 | 1.75E-01 | 2.41E-07 | 4.25E-01 | 0.00E+00 | 3.08E-12 | 6.16E-14 | 1.46E-03 | 3.21E-02 | 1.51E-03 | 1.16E-03 |
| Wind Speed | 6.03E-01 | 1.66E-01 | 7.40E-02 | 3.87E-13 | 6.73E-01 | 3.08E-12 | 0.00E+00 | 4.59E-27 | 1.20E-03 | 5.70E-02 | 6.15E-04 | 1.55E-04 |
| Wind Direction | 3.21E-01 | 3.47E-01 | 6.24E-01 | 1.02E-08 | 1.11E-01 | 6.16E-14 | 4.59E-27 | 0.00E+00 | 1.88E-04 | 9.88E-01 | 1.20E-08 | 1.31E-02 |
| Water Temperature | 5.09E-04 | 1.22E-02 | 2.40E-09 | 3.74E-14 | 2.65E-07 | 1.46E-03 | 1.20E-03 | 1.88E-04 | 0.00E+00 | 2.16E-09 | 4.10E-01 | 6.77E-02 |
| Dissolved Oxygen | 1.86E-10 | 1.48E-05 | 2.20E-11 | 1.86E-07 | 2.31E-13 | 3.21E-02 | 5.70E-02 | 9.88E-01 | 2.16E-09 | 0.00E+00 | 9.04E-07 | 1.30E-03 |
| Salinity | 1.78E-07 | 2.20E-02 | 1.04E-04 | 2.47E-01 | 2.15E-03 | 1.51E-03 | 6.15E-04 | 1.20E-08 | 4.10E-01 | 9.04E-07 | 0.00E+00 | 9.20E-01 |
| Turbidity | 4.71E-04 | 3.12E-03 | 3.25E-03 | 5.18E-03 | 9.05E-02 | 1.16E-03 | 1.55E-04 | 1.31E-02 | 6.77E-02 | 1.30E-03 | 9.20E-01 | 0.00E+00 |

`
