# Supplementary material for: Using a combination of quantitative culture, molecular, and infrastructure data to rank potential sources of fecal contamination in Town Creek Estuary, North Carolina
Source: PLoS One. 2024 Apr 19;19(4):e0299254. doi: 10.1371/journal.pone.0299254 (PMC11029655; doi:10.1371/journal.pone.0299254)
Supplement: S6 Table — ND indicates sample concentration below the limit of detection for HF183. (DOCX) [file pone.0299254.s007.docx]

**S6 Table**. Concentrations of HF183 (copies per 100 mL) and extraction recovery percentage for all sites and the method blank over the course of the project. ND indicates sample concentration below the limit of detection for HF183.

| Site | Site Description | Collection Date | HF 183 (Copies per 100 mL) | Lower 95% Confidence Interval (Copies per 100 mL) | Upper 95% Confidence Interval (Copies per 100 mL) | Extraction Recovery % |
| --- | --- | --- | --- | --- | --- | --- |
| 1 | Ace Hardware | 8/6/2021 | 123 | 50 | 253 | 53 |
| 2 | Channel-Ace Hardware | 8/6/2021 | ND | 0 | 97 | 32 |
| 3 | Stanton Road Finger | 8/6/2021 | ND | 0 | 63 | 27 |
| 4 | Stanton Road Channel | 8/6/2021 | ND | 0 | 70 | 36 |
| 5 | Marsh Finger | 8/6/2021 | ND | 0 | 73 | 32 |
| 6 | Stormwater Ditch Finger | 8/6/2021 | ND | 0 | 73 | 29 |
| 7 | Channel Under Turner Street Bridge | 8/6/2021 | ND | 0 | 77 | 32 |
| 8 | Town Creek Lift Station | 8/6/2021 | ND | 0 | 87 | 31 |
| 9 | Public Access Dock | 8/6/2021 | ND | 0 | 87 | 31 |
| 10 | Town Creek Marina | 8/6/2021 | 87 | 20 | 227 | 34 |
| 11 | Method Blank | 8/6/2021 | ND | 0 | 83 | 27 |
| 1 | Ace Hardware | 8/13/2021 | ND | 0 | 37 | 65 |
| 2 | Channel-Ace Hardware | 8/13/2021 | ND | 0 | 33 | 32 |
| 3 | Stanton Road Finger | 8/13/2021 | ND | 0 | 63 | 30 |
| 4 | Stanton Road Channel | 8/13/2021 | ND | 0 | 33 | 12 |
| 5 | Marsh Finger | 8/13/2021 | ND | 0 | 37 | 13 |
| 6 | Stormwater Ditch Finger | 8/13/2021 | ND | 0 | 31 | 33 |
| 7 | Channel Under Turner Street Bridge | 8/13/2021 | ND | 10 | 177 | 15 |
| 8 | Town Creek Lift Station | 8/13/2021 | ND | 0 | 77 | 28 |
| 9 | Public Access Dock | 8/13/2021 | ND | 0 | 90 | 17 |
| 10 | Town Creek Marina | 8/13/2021 | ND | 0 | 87 | 19 |
| 11 | Method Blank | 8/13/2021 | ND | 0 | 90 | 15 |
| 1 | Ace Hardware | 8/27/2021 | ND | 10 | 100 | 39 |
| 2 | Channel-Ace Hardware | 8/27/2021 | ND | 0 | 33 | 35 |
| 3 | Stanton Road Finger | 8/27/2021 | ND | 0 | 60 | 40 |
| 4 | Stanton Road Channel | 8/27/2021 | 37 | 3 | 70 | 16 |
| 5 | Marsh Finger | 8/27/2021 | ND | 0 | 53 | 15 |
| 6 | Stormwater Ditch Finger | 8/27/2021 | ND | 0 | 32 | 36 |
| 7 | Channel Under Turner Street Bridge | 8/27/2021 | ND | 0 | 123 | 12 |
| 8 | Town Creek Lift Station | 8/27/2021 | ND | 0 | 73 | 48 |
| 9 | Public Access Dock | 8/27/2021 | ND | 0 | 67 | 14 |
| 10 | Town Creek Marina | 8/27/2021 | ND | 0 | 67 | 20 |
| 11 | Method Blank | 8/27/2021 | ND | 0 | 67 | 13 |
| 1 | Ace Hardware | 9/10/2021 | ND | 0 | 37 | 49 |
| 2 | Channel-Ace Hardware | 9/10/2021 | ND | 0 | 33 | 36 |
| 3 | Stanton Road Finger | 9/10/2021 | ND | 0 | 33 | 40 |
| 4 | Stanton Road Channel | 9/10/2021 | ND | 0 | 32 | 16 |
| 5 | Marsh Finger | 9/10/2021 | ND | 0 | 37 | 16 |
| 6 | Stormwater Ditch Finger | 9/10/2021 | ND | 0 | 33 | 17 |
| 7 | Channel Under Turner Street Bridge | 9/10/2021 | ND | 0 | 40 | 15 |
| 8 | Town Creek Lift Station | 9/10/2021 | ND | 0 | 32 | 15 |
| 9 | Public Access Dock | 9/10/2021 | ND | 0 | 67 | 11 |
| 10 | Town Creek Marina | 9/10/2021 | ND | 0 | 90 | 11 |
| 11 | Method Blank | 9/10/2021 | ND | 0 | 77 | 10 |
| 1 | Ace Hardware | 9/20/2021 | ND | 0 | 53 | 12 |
| 2 | Channel-Ace Hardware | 9/20/2021 | ND | 0 | 32 | 13 |
| 3 | Stanton Road Finger | 9/20/2021 | ND | 0 | 33 | 13 |
| 4 | Stanton Road Channel | 9/20/2021 | ND | 0 | 32 | 12 |
| 5 | Marsh Finger | 9/20/2021 | ND | 0 | 37 | 11 |
| 6 | Stormwater Ditch Finger | 9/20/2021 | ND | 0 | 33 | 15 |
| 7 | Channel Under Turner Street Bridge | 9/20/2021 | ND | 0 | 32 | 17 |
| 8 | Town Creek Lift Station | 9/20/2021 | ND | 0 | 53 | 19 |
| 9 | Public Access Dock | 9/20/2021 | ND | 0 | 33 | 13 |
| 10 | Town Creek Marina | 9/20/2021 | ND | 0 | 33 | 14 |
| 11 | Method Blank | 9/20/2021 | ND | 0 | 33 | 14 |
| 1 | Ace Hardware | 9/24/2021 | ND | 0 | 31 | 12 |
| 2 | Channel-Ace Hardware | 9/24/2021 | ND | 0 | 30 | 13 |
| 3 | Stanton Road Finger | 9/24/2021 | ND | 0 | 47 | 18 |
| 4 | Stanton Road Channel | 9/24/2021 | ND | 0 | 31 | 12 |
| 5 | Marsh Finger | 9/24/2021 | ND | 0 | 33 | 15 |
| 6 | Stormwater Ditch Finger | 9/24/2021 | ND | 0 | 33 | 15 |
| 7 | Channel Under Turner Street Bridge | 9/24/2021 | ND | 0 | 33 | 13 |
| 8 | Town Creek Lift Station | 9/24/2021 | ND | 0 | 33 | 11 |
| 9 | Public Access Dock | 9/24/2021 | ND | 0 | 30 | 14 |
| 10 | Town Creek Marina | 9/24/2021 | ND | 0 | 29 | 19 |
| 11 | Method Blank | 9/24/2021 | ND | 0 | 32 | 11 |
| 1 | Ace Hardware | 9/28/2021 | 30 | 7 | 77 | 15 |
| 2 | Channel-Ace Hardware | 9/28/2021 | ND | 0 | 29 | 16 |
| 3 | Stanton Road Finger | 9/28/2021 | ND | 0 | 32 | 12 |
| 4 | Stanton Road Channel | 9/28/2021 | ND | 3 | 70 | 9 |
| 5 | Marsh Finger | 9/28/2021 | ND | 0 | 33 | 13 |
| 6 | Stormwater Ditch Finger | 9/28/2021 | ND | 0 | 33 | 15 |
| 7 | Channel Under Turner Street Bridge | 9/28/2021 | ND | 0 | 32 | 14 |
| 8 | Town Creek Lift Station | 9/28/2021 | ND | 0 | 30 | 15 |
| 9 | Public Access Dock | 9/28/2021 | ND | 0 | 31 | 15 |
| 10 | Town Creek Marina | 9/28/2021 | ND | 0 | 30 | 14 |
| 11 | Method Blank | 9/28/2021 | ND | 0 | 29 | 10 |
| 1 | Ace Hardware | 10/11/2021 | 183 | 110 | 283 | 18 |
| 2 | Channel-Ace Hardware | 10/11/2021 | 40 | 13 | 93 | 15 |
| 3 | Stanton Road Finger | 10/11/2021 | ND | 0 | 30 | 14 |
| 4 | Stanton Road Channel | 10/11/2021 | ND | 0 | 37 | 14 |
| 5 | Marsh Finger | 10/11/2021 | ND | 0 | 32 | 15 |
| 6 | Stormwater Ditch Finger | 10/11/2021 | ND | 0 | 33 | 16 |
| 7 | Channel Under Turner Street Bridge | 10/11/2021 | ND | 0 | 32 | 17 |
| 8 | Town Creek Lift Station | 10/11/2021 | ND | 0 | 33 | 16 |
| 9 | Public Access Dock | 10/11/2021 | ND | 3 | 70 | 16 |
| 10 | Town Creek Marina | 10/11/2021 | ND | 0 | 33 | 13 |
| 11 | Method Blank | 10/11/2021 | ND | 0 | 33 | 13 |
