# Supplementary material for: Using a combination of quantitative culture, molecular, and infrastructure data to rank potential sources of fecal contamination in Town Creek Estuary, North Carolina
Source: PLoS One. 2024 Apr 19;19(4):e0299254. doi: 10.1371/journal.pone.0299254 (PMC11029655; doi:10.1371/journal.pone.0299254)
Supplement: S7 Table — a. Spearman rank correlation test statistics (rs) between fecal indicator bacteria (FIB) species with piping materials and construction dates. b. Spearman rank correlation test p-values between fecal indicator bacteria (FIB) species with piping materials and construction dates. (DOCX) [file pone.0299254.s008.docx]

**S7a Table**. Spearman rank correlation test statistics (r_s_) between fecal indicator bacteria (FIB) species with piping materials and construction dates. TC= total coliforms, EC= *Escherichia coli*, ENT=*Enterococcus,* VC= vitrified clay, PVC=polyvinyl chloride, DIP=ductile iron pipe, CIPP=cured in place pipe, Age > 50= percentage of pipe at each site greater than 50 years of age, Age <50= percentage of pipe at each site less than 50 years of age

|  | TC | EC | ENT | VC | Truss | PVC | DIP | CIPP | Age > 50 | Age < 50 |
| --- | --- | --- | --- | --- | --- | --- | --- | --- | --- | --- |
| TC | 1 | 0.72*** | 0.83*** | 0.54*** | 0.59*** | -0.45*** | -0.59*** | 0.44*** | 0.54*** | -0.54*** |
| EC | 0.72*** | 1 | 0.58*** | 0.52*** | 0.58*** | -0.42*** | -0.55*** | 0.42*** | 0.52*** | -0.52*** |
| ENT | 0.83*** | 0.58*** | 1 | 0.48*** | 0.54*** | -0.44*** | -0.52*** | 0.41*** | 0.48*** | -0.48*** |
| VC | 0.54*** | 0.52*** | 0.48*** | 1 | 0.69*** | -0.93*** | -0.98*** | 0.94*** | 1 | -1 |
| Truss | 0.59*** | 0.58*** | 0.54*** | 0.69*** | 1 | -0.7*** | -0.68*** | 0.69*** | 0.69*** | -0.69*** |
| PVC | -0.45*** | -0.42*** | -0.44*** | -0.93*** | -0.7*** | 1 | 0.9*** | -0.98*** | -0.93*** | 0.93*** |
| DIP | -0.59*** | -0.55*** | -0.52*** | -0.98*** | -0.68*** | 0.9*** | 1 | -0.91*** | -0.98*** | 0.98*** |
| CIPP | 0.44*** | 0.42*** | 0.41*** | 0.94*** | 0.69*** | -0.98*** | -0.91*** | 1 | 0.94*** | -0.94*** |
| Age > 50 | 0.54*** | 0.52*** | 0.48*** | 1 | 0.69*** | -0.93*** | -0.98*** | 0.94*** | 1 | -1 |
| Age < 50 | -0.54*** | -0.52*** | -0.48*** | -1 | -0.69*** | 0.93*** | 0.98*** | -0.94*** | -1 | 1 |

(* = p < 0.05, ** = p < 0.01, and *** = p < 0.001)

**S7b Table**. Spearman rank correlation test p-values between fecal indicator bacteria (FIB) species with piping materials and construction dates. TC=total coliforms, EC= *Escherichia coli*, ENT=*Enterococcus,* VC= vitrified clay, PVC=polyvinyl chloride, DIP=ductile iron pipe, CIPP=cured in place pipe, Age > 50= percentage of pipe at each site greater than 50 years of age, Age <50= percentage of pipe at each site less than 50 years of age

|  | _TC_ | _EC_ | _ENT_ | _VC_ | _Truss_ | _PVC_ | _DIP_ | _CIPP_ | _Age > 50_ | _Age < 50_ |
| --- | --- | --- | --- | --- | --- | --- | --- | --- | --- | --- |
| _TC_ | 0.00E+00 | 9.60E-15 | 1.46E-18 | 1.98E-07 | 2.79E-08 | 2.07E-05 | 1.26E-08 | 5.26E-05 | 1.98E-07 | 1.98E-07 |
| _EC_ | 9.60E-15 | 0.00E+00 | 1.34E-07 | 2.34E-07 | 1.22E-08 | 4.05E-05 | 2.08E-08 | 3.91E-05 | 2.34E-07 | 2.34E-07 |
| _ENT_ | 1.46E-18 | 1.34E-07 | 0.00E+00 | 3.10E-05 | 1.33E-06 | 1.56E-04 | 5.03E-06 | 4.51E-04 | 3.10E-05 | 3.10E-05 |
| _VC_ | 1.98E-07 | 2.34E-07 | 3.10E-05 | 0.00E+00 | 1.87E-12 | 1.44E-35 | 5.74E-58 | 4.66E-37 | 0.00E+00 | 0.00E+00 |
| _Truss_ | 2.79E-08 | 1.22E-08 | 1.33E-06 | 1.87E-12 | 0.00E+00 | 3.69E-13 | 2.87E-12 | 2.32E-12 | 1.87E-12 | 1.87E-12 |
| _PVC_ | 2.07E-05 | 4.05E-05 | 1.56E-04 | 1.44E-35 | 3.69E-13 | 0.00E+00 | 8.29E-30 | 5.97E-58 | 1.44E-35 | 1.44E-35 |
| _DIP_ | 1.26E-08 | 2.08E-08 | 5.03E-06 | 5.74E-58 | 2.87E-12 | 8.29E-30 | 0.00E+00 | 8.14E-31 | 5.74E-58 | 5.74E-58 |
| _CIPP_ | 5.26E-05 | 3.91E-05 | 4.51E-04 | 4.66E-37 | 2.32E-12 | 5.97E-58 | 8.14E-31 | 0.00E+00 | 4.66E-37 | 4.66E-37 |
| _Age > 50_ | 1.98E-07 | 2.34E-07 | 3.10E-05 | 0.00E+00 | 1.87E-12 | 1.44E-35 | 5.74E-58 | 4.66E-37 | 0.00E+00 | 0.00E+00 |
| _Age < 50_ | 1.98E-07 | 2.34E-07 | 3.10E-05 | 0.00E+00 | 1.87E-12 | 1.44E-35 | 5.74E-58 | 4.66E-37 | 0.00E+00 | 0.00E+00 |
