# Supplementary material for: Using a combination of quantitative culture, molecular, and infrastructure data to rank potential sources of fecal contamination in Town Creek Estuary, North Carolina
Source: PLoS One. 2024 Apr 19;19(4):e0299254. doi: 10.1371/journal.pone.0299254 (PMC11029655; doi:10.1371/journal.pone.0299254)
Supplement: S8 Table — a. Rank results for each measured parameter using the equal weighting method. b. Rank results for each measured parameter using the variable weighting method. (DOCX) [file pone.0299254.s009.docx]

**S8a Table.** Rank results for each measured parameter using the equal weighting method.

| Site | Vitrified Clay Pipes (%) | Rank* | Pipes aged over 50 years (%) | Rank* | Mean EC (MPN per 100 mL) | Rank* | Mean ENT (MPN per 100mL) | Rank* | Mean HF183 (Copies per 100mL) | Rank* | Approximate Distance to Nearest Stormwater Pipe (m)+ | Rank* | Rank Sum Total | Rank Using Equal Weighting |
| --- | --- | --- | --- | --- | --- | --- | --- | --- | --- | --- | --- | --- | --- | --- |
| 1 | 32% | 9 | 64% | 9 | 2907.6 | 8 | 4504.3 | 10 | 42.0 | 10 | 60 | 7 | 53 | 1 |
| 2 | 47% | 10 | 65% | 10 | 2588.9 | 7 | 1093.9 | 9 | 5.0 | 8 | 80 | 5 | 49 | 2 |
| 3 | 29% | 8 | 37% | 8 | 513.4 | 6 | 284.7 | 8 | 0.0 | 1 | 100 | 2 | 33 | 4^#^ |
| 4 | 26% | 7 | 33% | 7 | 308.4 | 4 | 71.5 | 5 | 4.6 | 7 | 100 | 2 | 32 | 6 |
| 5 | 26% | 7 | 33% | 7 | 282.4 | 3 | 77.4 | 6 | 0.0 | 1 | 60 | 7 | 31 | 7 |
| 6 | 19% | 5 | 25% | 5 | 6429.6 | 10 | 256.4 | 7 | 0.0 | 1 | 5 | 10 | 38 | 3 |
| 7 | 19% | 5 | 25% | 5 | 3331.0 | 9 | 69.4 | 4 | 0.0 | 1 | 15 | 9 | 33 | 4^#^ |
| 8 | 14% | 2 | 18% | 2 | 404.2 | 5 | 57.8 | 3 | 0.0 | 1 | 20 | 8 | 21 | 8 |
| 9 | 19% | 5 | 19% | 3 | 200.4 | 1 | 37.4 | 2 | 0.0 | 1 | 95 | 4 | 16 | 9 |
| 10 | 0% | 1 | 0% | 1 | 235.3 | 2 | 12.3 | 1 | 10.9 | 9 | 160 | 1 | 15 | 10 |

*****Rank = sites are ranked 1-10 based on the mean value of the measured parameter with 10 representing the highest mean and 1 representing the lowest mean, with the exception of +(approximate distance to nearest stormwater pipe) in which the shortest distance to the pipe received the highest rank of 10 and the furthest distance received the lowest weight of 1.

#Using the equal weighting method, Sites 3 and 7 were equally ranked as the 4^th^ most likely to contribute to fecal contamination in the estuary; the remaining site rankings were adjusted accordingly (i.e., the next highest ranked site received a score of 6).

**S8b Table.** Rank results for each measured parameter using the variable weighting method.

| Parameter Weight | 1 |  | 1 |  | 4 |  | 4 |  | 6 |  | 1 |  |  |  |
| --- | --- | --- | --- | --- | --- | --- | --- | --- | --- | --- | --- | --- | --- | --- |
| Site | **Vitrified Clay Pipes (%)** | **Rank (Weight X Mean Rank*)** | **Pipes aged over 50 years (%)** | **Rank (Weight X Mean Rank*)** | **Mean EC (MPN per 100 mL)** | **Rank (Weight X Mean Rank*)** | **Mean ENT (MPN per 100mL)** | **Rank (Weight X Mean Rank*)** | **Mean HF183 (Copies per 100mL)** | **Rank (Weight X Mean Rank*)** | **Approximate Distance to Nearest Stormwater Pipe (m)** | **Rank (Weight X Mean Rank*)** | **Rank Sum Total** | **Rank Using Variable Weighting** |
| 1 | 32% | 9 | 64% | 9 | 2907.6 | 32 | 4504.3 | 40 | 42.0 | 60 | 60 | 7 | 157 | 1 |
| 2 | 47% | 10 | 65% | 10 | 2588.9 | 28 | 1093.9 | 36 | 5.0 | 48 | 80 | 5 | 137 | 2 |
| 3 | 29% | 8 | 37% | 8 | 513.4 | 24 | 284.7 | 32 | 0.0 | 6 | 100 | 2 | 80 | 5 |
| 4 | 26% | 7 | 33% | 7 | 308.4 | 16 | 71.5 | 20 | 4.6 | 42 | 100 | 2 | 94 | 3^#^ |
| 5 | 26% | 7 | 33% | 7 | 282.4 | 12 | 77.4 | 24 | 0.0 | 6 | 60 | 7 | 63 | 8 |
| 6 | 19% | 5 | 25% | 5 | 6429.6 | 40 | 256.4 | 28 | 0.0 | 6 | 5 | 10 | 94 | 3^#^ |
| 7 | 19% | 5 | 25% | 5 | 3331.0 | 36 | 69.4 | 16 | 0.0 | 6 | 15 | 9 | 77 | 6 |
| 8 | 14% | 2 | 18% | 2 | 404.2 | 20 | 57.8 | 12 | 0.0 | 6 | 20 | 8 | 50 | 9 |
| 9 | 19% | 5 | 19% | 3 | 200.4 | 4 | 37.4 | 8 | 0.0 | 6 | 95 | 4 | 30 | 10 |
| 10 | 0% | 1 | 0% | 1 | 235.3 | 8 | 12.3 | 4 | 10.9 | 54 | 160 | 1 | 69 | 7 |

*Mean rank refers to the value of each site ranked 1-10 based on the mean value of the measured parameter with 10 representing the highest mean and 1 representing the lowest mean, with the exception of +(approximate distance to nearest stormwater pipe) in which the shortest distance to the pipe received the highest rank of 10 and the furthest distance received the lowest weight of 1.

#Using the variable weighting method, Sites 3 and 6 were equally ranked as the 3^rd^ most likely to contribute to fecal contamination in the estuary; the remaining site rankings were adjusted accordingly (i.e., the next highest ranked site received a score of 5).
