# Supplementary material for: Using a combination of quantitative culture, molecular, and infrastructure data to rank potential sources of fecal contamination in Town Creek Estuary, North Carolina
Source: PLoS One. 2024 Apr 19;19(4):e0299254. doi: 10.1371/journal.pone.0299254 (PMC11029655; doi:10.1371/journal.pone.0299254)
Supplement: S1 Method — (DOCX) [file pone.0299254.s011.docx]

**S1 Method:** Design and sequence of the microbial source tracking control.

HF183 positive control was synthesized as a GeneBlock (IDT, Coralville, IA) using a 299 nucleotide fragment (bases 121-420) of *Bacteroides dorei* strain 175 16S ribosomal RNA gene, partial sequence NCBI Reference Sequence: NR_041351.1. The GeneBlock was resuspended in Buffer AE (QIAGEN, Germantown, MD) and frozen at -80ºC in single use aliquots. The copy number was determined via ddPCR quantification.

121 atccaacctg ccgtctactc ttggccagcc ttctgaaagg aagattaatc caggatggga

181 tcatgagttc acatgtccgc atgattaaag gtattttccg gtagacgatg gggatgcgtt

241 ccattagata gtaggcgggg taacggccca cctagtcaac gatggatagg ggttctgaga

301 ggaaggtccc ccacattgga actgagacac ggtccaaact cctacgggag gcagcagtga

361 ggaatattgg tcaatgggcg atggcctgaa ccagccaagt agcgtgaagg atgactgccc

For preparation of the *gyrA* inhibition control, halophilic archaeon cells were lysed at 100ºC for 5 minutes, followed by a 5 minute spin at 10,000 x g. The supernatant was removed, and single use aliquots were stored frozen at -20ºC. Cell lysate was quantified for the *gyrA* gene via ddPCR. The primer and probe sequences for this proprietary assay are in preparation for publication. For additional information about this assay, please contact:

Joshua A. Steele, joshuas@sccwrp.org

John F. Griffith, johng@sccwrp.org

1 Southern California Coastal Water Research Project, Costa Mesa, CA, USA
