# Supplementary material for: Using a combination of quantitative culture, molecular, and infrastructure data to rank potential sources of fecal contamination in Town Creek Estuary, North Carolina
Source: PLoS One. 2024 Apr 19;19(4):e0299254. doi: 10.1371/journal.pone.0299254 (PMC11029655; doi:10.1371/journal.pone.0299254)
Supplement: S2 Method — (DOCX) [file pone.0299254.s012.docx]

**S2 Method**: Two parameter weighting methods for ranking potential sources of fecal contamination in Town Creek Estuary, Beaufort, North Carolina.

**Equal Weighting**

Using the equal weighting method, each of the six parameters included in the method (percentage of pipes in a 400-m radius made of vitrified clay, percentage of pipes in a 400-m radius over 50 years of age, the inverse of the distance to the nearest stormwater pipe in a 400-m radius, mean *Escherichia coli* concentration, mean *Enterococcus concentration*, and mean HF183 concentration) were given equal weight. Each parameter was ranked from one to ten at each site, with ten being the site with the highest mean value for each parameter and one being the site with the lowest mean value for each parameter. The rank for each site for each parameter was then summed and a total was scored for each site. The site with the highest overall score was ranked the highest potential source of fecal contamination while the site with the lowest overall score was ranked as the lowest potential source of fecal contamination in the estuary. Actual ranks for each parameter based on the mean parameter value can be found in S8a Table above.

**Variable Weighting**

Using the variable weighting method, each of the six parameters included in the method (percentage of pipes in a 400-m radius made of vitrified clay, percentage of pipes in a 400-m radius over 50 years of age, the inverse of the distance to the nearest stormwater pipe in a 400-m radius, mean *Escherichia coli* concentration, mean *Enterococcus concentration*, and mean HF183 concentration) were given a variable weight based on empirical knowledge of the parameter. Parameters were weighted 1-6, with 6 being the highest weight, based on whether or not the presence of the parameter indicated the presence of human fecal contamination, with those parameters directly linking fecal contamination to human sources ranked the highest weight. If a parameter had an equal likelihood of indicating the presence of fecal contamination as another parameter, they were given the same weight and the values of subsequent weight were adjusted accordingly.

**Weights for each parameter were assigned as follows:**

| Parameter | Weight* |
| --- | --- |
| HF183 | 6 |
| *Escherichia coli* | 4 |
| *Enterococcus* | 4 |
| Percentage of Vitrified Clay Pipe | 1 |
| Percentage of Pipe Aged over 50 years | 1 |
| Inverse distance to nearest stormwater pipe | 1 |

HF183 is a molecular marker specific to human fecal contamination, thus it received a weight of six. Both *Escherichia coli* and *Enterococcus* are fecal bacterial indicators, thus they received an equal weighting of 4. The remaining three sewage infrastructure parameters are indirect indicators of potential sources of fecal contamination, thus they each received a weight of 1.

Each parameter weight was multiplied by the rank of the parameter at each site (again, ranked from one to ten, with ten being the site with the highest mean value for each parameter and one being the site with the lowest mean value for each parameter). These values were summed across each site and a total was scored. The site with the highest overall score was ranked the highest potential source of fecal contamination while the site with the lowest overall score was ranked as the lowest potential source of fecal contamination in the estuary. The exact weight and rank for each site can be found in Table S8b above.
