# Supplementary material for: Using a combination of quantitative culture, molecular, and infrastructure data to rank potential sources of fecal contamination in Town Creek Estuary, North Carolina
Source: PLoS One. 2024 Apr 19;19(4):e0299254. doi: 10.1371/journal.pone.0299254 (PMC11029655; doi:10.1371/journal.pone.0299254)
Supplement: S1 Equation — (DOCX) [file pone.0299254.s013.docx]

**S1 Equation.** Equation to calculate gene copies/L from ddPCR (originally published in Beattie *et al.* 2022[1]).

To calculate X gene copies/100 mL, the machine generated copies/µL (A) is multiplied by the total volume of the duplicated PCR reaction (here, 25 µL/ reaction * 2 = 50 µL) divided by the volume of eluted nucleic acids in the PCR reaction (here, 10 µL), multiplied by the total nucleic extraction elution volume (here, 100 µL), then divided by the ratio of 100 mL to volume filtered (here, 150 mL).

$$\text{X }\frac{copies}{L} = \text{A}\frac{copies}{\mu L} \times\frac{50 \mu L}{10 \mu L} \times100 \mu L \div\frac{100 mL}{150 mL}$$

**References**

1. Beattie RE, Blackwood AD, Clerkin T, Dinga C, Noble RT. Evaluating the impact of sample storage, handling, and technical ability on the decay and recovery of SARS-CoV-2 in wastewater. PLOS ONE. 2022 Jun 24;17(6):e0270659.
